# Resistance to PARP inhibitors by SLFN11 inactivation can be overcome by ATR inhibition

**DOI:** 10.18632/oncotarget.12266

**Published:** 2016-09-27

**Authors:** Junko Murai, Ying Feng, Guoying K. Yu, Yuanbin Ru, Sai-Wen Tang, Yuqiao Shen, Yves Pommier

**Affiliations:** ^1^ Developmental Therapeutics Branch and Laboratory of Molecular Pharmacology, Center for Cancer Research, National Cancer Institute, National Institutes of Health, Bethesda, MD, USA; ^2^ BioMarin Pharmaceutical Inc., Novato, CA, USA; ^3^ Current affiliation: Division of Blood and Marrow Transplantation, Department of Medicine, Stranford University School of Medicine, Stanford, CA, USA

**Keywords:** PARP-trapping, ATR, PARP inhibitor, BRCA, homologous recombination

## Abstract

Poly(ADP-ribose) polymerase inhibitors (PARPIs) kill cancer cells by trapping PARP1 and PARP2. Talazoparib, the most potent PARPI inhibitor (PARPI), exhibits remarkable selectivity among the NCI-60 cancer cell lines beyond BRCA inactivation. Our genomic analyses reveal high correlation between response to talazoparib and *Schlafen 11* (*SLFN11*) expression. Causality was established in four isogenic *SLFN11*-positive and -negative cell lines and extended to olaparib. Response to the talazoparib-temozolomide combination was also driven by SLFN11 and validated in 36 small cell lung cancer cell lines, and in xenograft models. Resistance in *SLFN11*-deficient cells was caused neither by impaired drug penetration nor by activation of homologous recombination. Rather, SLFN11 induced irreversible and lethal replication inhibition, which was independent of ATR-mediated S-phase checkpoint. The resistance to PARPIs by SLFN11 inactivation was overcome by ATR inhibition, mechanistically because *SLFN11*-deficient cells solely rely on ATR activation for their survival under PARPI treatment. Our study reveals that SLFN11 inactivation, which is common (~45%) in cancer cells, is a novel and dominant resistance determinant to PARPIs.

## INTRODUCTION

Several PARP inhibitors (PARPIs) are in advanced clinical trials, and olaparib has recently been approved for advanced ovarian cancer carrying BRCA mutations. All clinical PARPIs are competitive NAD^+^ inhibitors. They all block poly(ADP-ribosyl)ation (PARylation) [1, 2], which is a critical step of base excision repair (BER), the major pathway for repairing DNA single-strand breaks (SSBs) [3, 4]. Since SSBs are among the most frequent endogenous DNA lesions repaired by PARP1 and PARP2, and the discovery of the synthetic lethality of PARP inhibitors in BRCA-deficient cells, the mechanism by which PARPIs exert their cytotoxicity has been dominantly interpreted as an accumulation of SSBs resulting in lethal DNA double-strand breaks upon replication stalling in cancer cells defective in homologous recombination (HR) [5, 6]. Further studies showed that PARPIs with equivalent potency as PARylation inhibitors had widely different cytotoxicity [2, 7, 8], and that this differential cytotoxicity was driven by the potency of the drugs to stabilize PARP-DNA complexes at SSBs (PARP-trapping) [7, 8]. Hence, the clinical PARPIs differ by their PARP trapping potency (talazoparib >> niraparib ≈ olaparib ≈ rucaparib >> veliparib), which corresponds to their cytotoxic potency [9].

PARP-DNA complexes can be obstacles for replication and induce replicative DNA damage [8]. In response to replicative damage, ATR (ataxia telangiectasia and Rad3-related protein kinase) plays a major role for coordinating cell cycle progression and DNA repair [10, 11]. ATR activates the S-phase checkpoint by phosphorylating the cell cycle checkpoint kinase 1, CHK1 at serine 345, which slows down replication forks (elongation checkpoint), stabilizes stalled replication forks and prevents replication origin firing (origin firing checkpoint) [12-14]. The S-phase checkpoint promotes DNA repair and prevents premature mitosis, thereby maintaining genomic stability [10, 11]. Loss of the S-phase checkpoint by inhibitors of ATR or CHK1 causes unscheduled firing of replication origins in S-phase and the induction of DNA double-strand breaks [13, 15-17].

The US National Cancer Institute cancer cell lines (NCI-60), which are derived from 9 tissues of origin: breast, colon, skin, blood, central nervous system, lung, prostate, ovary and kidney, is the most annotated set of cancer cell lines with whole genome expression and mutation profiles, and drug responses for more than 200,000 compounds [18-20], as well as a variety of molecular and cellular processes [20, 21]. Taking advantage of the NCI-60, we previously discovered *Schlafen 11* (*SLFN11*) expression as an unanticipated genomic determinant of response to topoisomerase (Top) 1 inhibitors, Top2 inhibitors, alkylating agents, and DNA synthesis inhibitors [22-24]. Independently, SLFN11 was identified as a predictive genomic biomarker for Top1 inhibitors in the larger database of the Cancer Cell Line Encyclopedia (CCLE) [25]. Importantly, lack of *SLFN11* mRNA expression is observed in ~45% of the cancer cells in the NCI-60 and CCLE panel. The importance of SLFN11 for drug sensitivity has recently been extended to Ewing's sarcomas [26], and to patient responses in ovarian, non-small cell lung and colorectal cancers [23, 24, 27]. A recent study revealed that SLFN11 inhibits checkpoint maintenance and homologous recombination by removing RPA from single stranded DNA [28].

**Figure 1:**
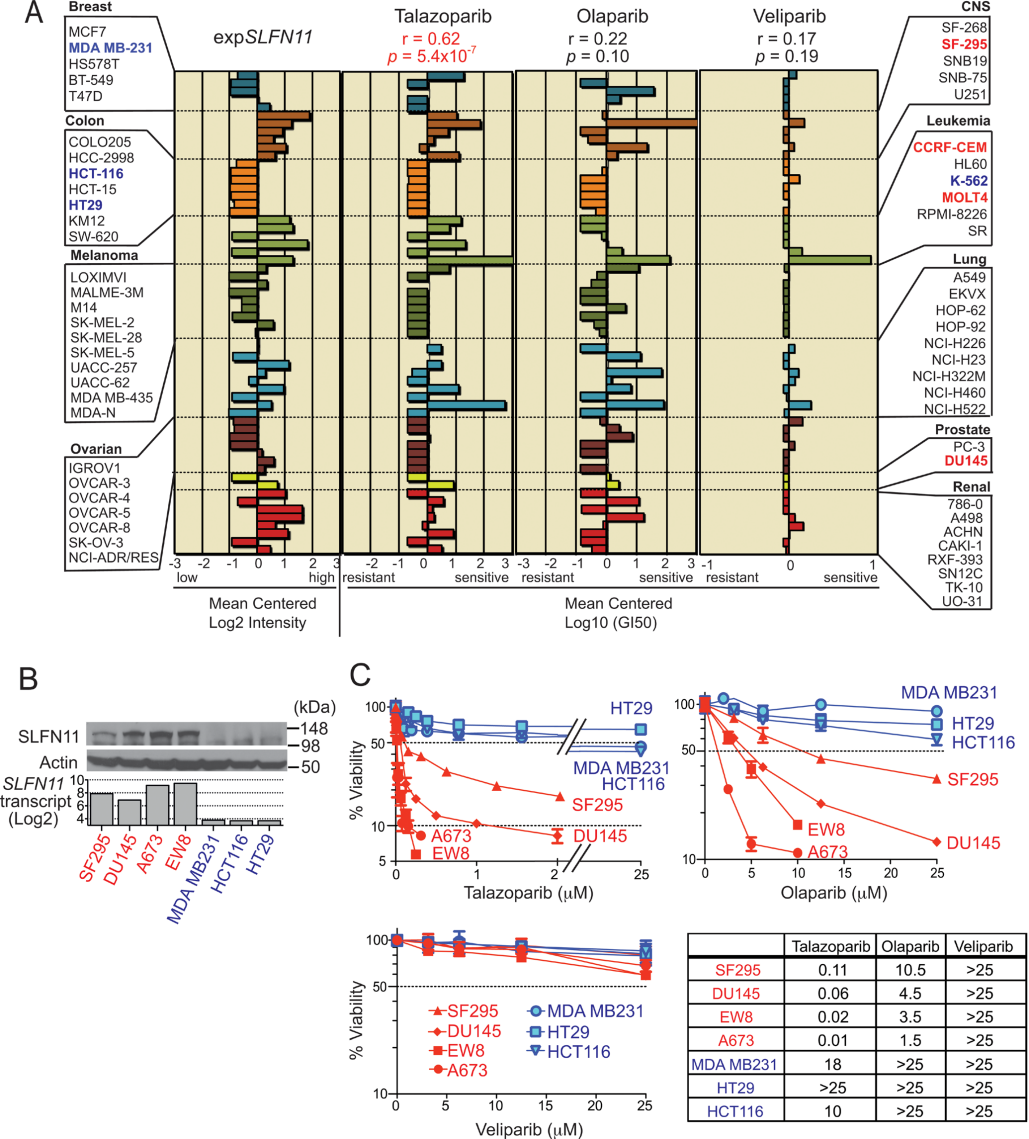
*SLFN11* expression is highly correlated with sensitivity to talazoparib **A.** Mean-centered bar charts [20] representing *SLFN11* expression (left), and sensitivity to talazoparib (middle left), olaparib (middle right) and veliparib (right) in the NCI-60. Color codes correspond to tissue of origin annotated on the sides [20]. Pearson's correlation coefficient (*r*) and two-sided *P* value (*p*) between *SLFN11* transcripts and talazoparib or olaparib or veliparib are shown above each chart. The *SLFN11*-negative cell lines used for further analysis are in blue font (MDA_MB-231, HCT-116, HT29 and K-562), and the *SLFN11*-positive cell lines in red (SF-295, CCRF-CEM, MOLT4, and DU-145). **B.** Western blots of whole cell extract for the indicated cell lines and antibodies. Transcript level of *SLFN11* obtained from the NCI-60 (SF-295, DU145, MDA_MB-231, HCT-116 and HT29 cell lines) and the Cancer Cell Line Encyclopedia (EW8 and A673 cell lines) database in the indicated cell lines are shown with bar graph. **C.** Viability curves of the indicated cell lines after continuous treatment for 72 hours with the indicated PARPIs. ATPlite assay was used to measure cell viability. The viability of untreated cells was set as 100%. Error bars represent standard deviation (SD, *n* ≥ 3). Drug IC_90_ values μM are tabulated at the right bottom. EW8 and A673 are Ewing's sarcoma cell lines

Although talazoparib is the most potent PARPI for PARP-trapping [7, 9], approximately half of the NCI-60 cell lines are highly resistant to the drug, with cell viability above 50% even when the cells are treated with 100 μM talazoparib (~1,000-fold more than clinical relevant blood concentrations) [7]. On the other hand, about half of the cell lines are highly sensitive to talazoparib at low micromolar or nanomolar ranges of IC_50_ (inhibitory concentration 50%). Although BRCA status may affect the differential sensitivity in each cell line, BRCA deficiency by homozygous deleterious mutation or lack of expression is only found in one of the NCI-60 cell lines [22]. Moreover, this BRCA2-deficient cell line (HCC2998) is resistant to talazoparib [7] (Figure 1A). Therefore, uncovered determinants of response to talazoparib, olaparib and other PARPIs beyond BRCA are awaiting discovery. In this study, we demonstrate the importance of SLFN11 expression as a determinant of response to talazoparib in cancer cell lines and in xenograft models, and extend these findings to olaparib and to the combination of talazoparib with temozolomide. We also provide a rationale to overcome resistance to PARP inhibitors in *SLFN11*-negative cells by combining PARP and ATR inhibitors.

## RESULTS

### *SLFN11* expression correlates with sensitivity to PARP inhibitors

To identify novel genomic determinants of response to talazoparib, we took advantage of the fact that talazoparib (BMN 673) had been tested in the NCI-60 [7] and of the extensive NCI-60 genomic databases available through the Web application CellMiner (http://discover.nci.nih.gov/cellminer/) [20, 22]. *Schlafen 11* (*SLFN11*) came up as one of the top ranking genes (Pearson's *r* = 0.62, *p* = 5.4×10^−7^) (Figure 1A). The two other PARP inhibitors in the NCI-60 database, olaparib and veliparib, showed positive but not statistically significant correlation with *SLFN11* expression (Figure 1A, right panels).

The correlation between *SLFN11* expression and PARPI response was independently tested in five NCI-60 cells lines, two with high *SLFN11* transcripts, prostate DU145 and CNS SF295, and three with low transcripts, breast MDA_MB231, colon HT29 and HCT116. Additionally, we tested two Ewing's sarcoma cell lines, EW8 and A673 with high *SLFN11* transcripts [25, 26]. SLFN11 protein levels were consistent with transcript levels (Figure 1B). *SLFN11*-positive cells (red) were more sensitive to both talazoparib and olaparib with lower IC_50_ (inhibitory concentration 50%) than *SLFN11*-negative cells (blue) (Figure 1C). The differential sensitivity of *SLFN11*-positive vs. -negative cells was even more pronounced for talazoparib than olaparib. On the other hand, for veliparib, none of the cells reached IC_50_ at drug concentrations up to 25 μM. These results revealed that SLFN11 expression is correlated with the sensitivity to PARP-trapping inhibitors (olaparib and talazoparib) but not to the relatively pure catalytic PARP inhibitor (veliparib) [7, 9].

### Genetic inactivation of *SLFN11* renders cancer cells resistant to PARPIs

To determine the causal involvement of SLFN11 for PARPI sensitivity, we generated *SLFN11*-deleted (*SLFN11*-del) isogenic cell lines from four cell lines with high *SLFN11* (prostate DU145, leukemia CCRF-CEM and MOLT4, and Ewing's sarcoma EW8) [23, 26] using CRISPR/Cas9 (Figure S1). To avoid off-target effects by the similarity of guide RNA sequences to off-target genome regions, we designed two guide RNA sequences, (A) and (B), and generated independent clones using each guide RNA in every cell line. In the absence of drug treatment, there was no apparent difference in cell cycle or growth rate between the parental and *SLFN11*-del cells across the four cell lines (Figure S1).

**Figure 2:**
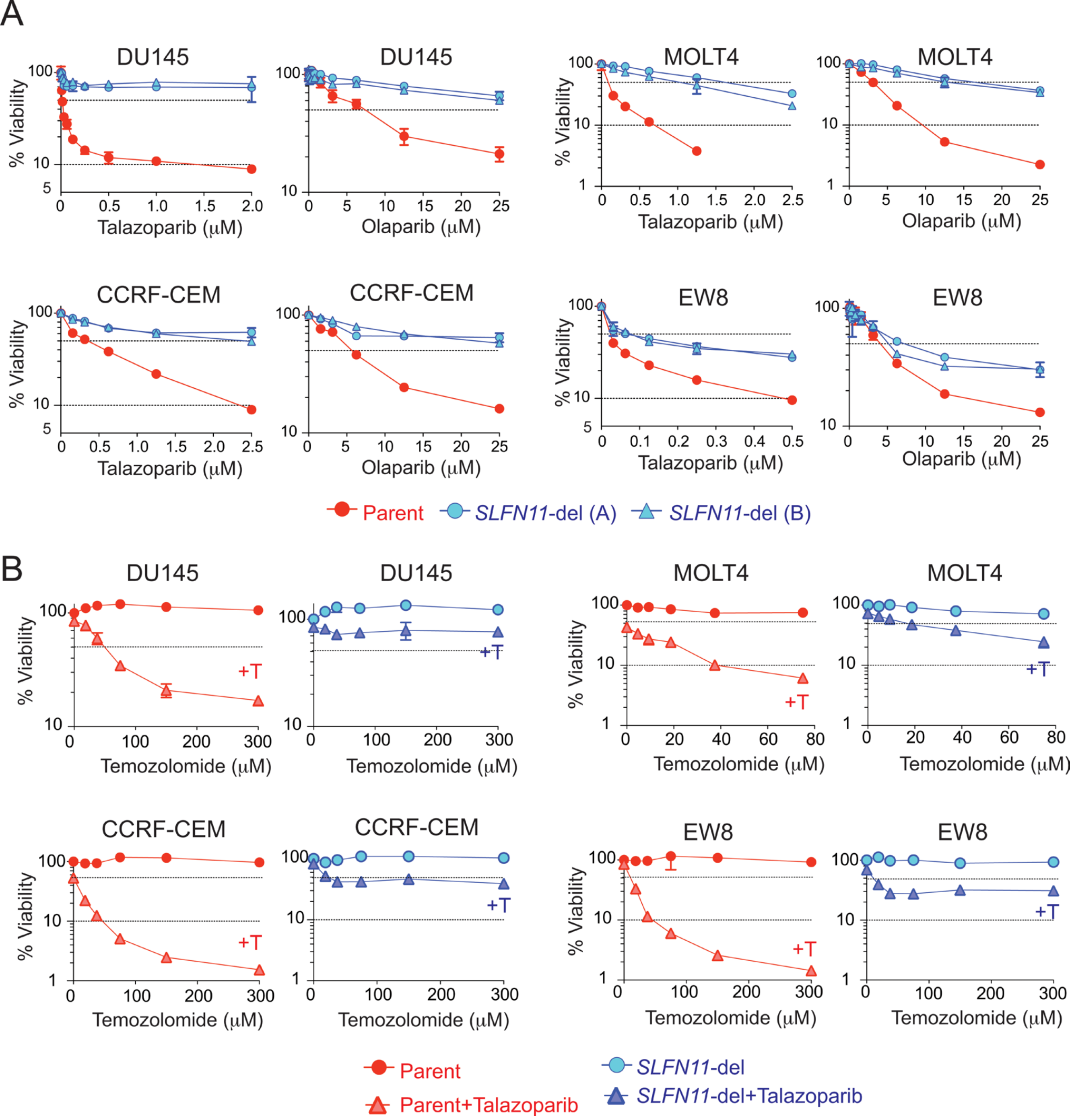
*SLFN11* inactivation confers resistance to talazoparib and olaparib **A.** Viability curves of the indicated parent and *SLFN11*-del cell lines in response to talazoparib or olaparib. Viability was determined as Figure 1C. Error bars represent SD (*n* ≥ 3). **B.** Viability curves of the indicated pairs of parental (red) and *SLFN11*-del (blue) cells treated with temozolomide alone (circle) or with temozolomide plus 10 nM talazoparib (+T, triangle). Viability of untreated cells was set as 100%. Error bars represent SD (*n* ≥ 3)

All four *SLFN11*-del cell lines showed resistance to both talazoparib and olaparib compared to their parental counterpart in 72 hours cell viability assays (Figure 2A). Consistent results were obtained by clonogenic assays (Figure S2A) and acute depletion of SLFN11 with siRNA transfection (Figure S2B). Depletion of SLFN11 by siRNA conferred as much resistance as depleting PARP1 itself, which mediates the cytotoxicity of talazoparib and olaparib [7, 8] (Figure S2B). Conversely, exogenous expression of SLFN11 in leukemia K562 cells that have very low *SLFN11* transcript (Figure 1A) conferred hypersensitivity to talazoparib and olaparib (Figure S2C). Hence, we conclude that *SLFN11* is a dominant determinant of sensitivity to PARP inhibitors.

Temozolomide, which is FDA-approved for glioblastomas, is highly synergistic with PARPIs even at concentrations where neither talazoparib nor temozolomide alone affect cell viability [7, 29]. This is because temozolomide alkylates guanine N7 resulting in abasic sites and single-strand breaks that recruit PARP1 and PARP2 and lead to PARP trapping [29]. Accordingly, combinations of PARP inhibitors and temozolomide are currently in clinical trials for various cancers beyond BRCA status [30]. We compared the talazoparib-temozolomide combination in the four isogenic parental and *SLFN11*-del cells (Figure 2B). The MGMT (O^6^-methylguanine DNA methyltransferase) status is known to determine temozolomide sensitivity [31, 32]. All four cell lines used for knocking out *SLFN11* are MGMT-proficient (data not shown), and therefore highly resistant to temozolomide because O6-methylguanine adducts are readily repaired by MGMT, and the DNA nicks generated by N7-methylguanine [32] are readily repaired in PARP1/2 proficient cells (Figure 2B). The addition of talazoparib markedly and synergistically sensitized the parental cells to temozolomide. However, in *SLFN11*-del cells, the combination had marginal effect (Figure 2B). These results demonstrate that *SLFN11* expression determines the sensitivity to PARP inhibitor-temozolomide combination in MGMT-proficient cells.

### *SLFN11* does not impact drug penetration or homologous recombination (HR) activation

**Figure 3:**
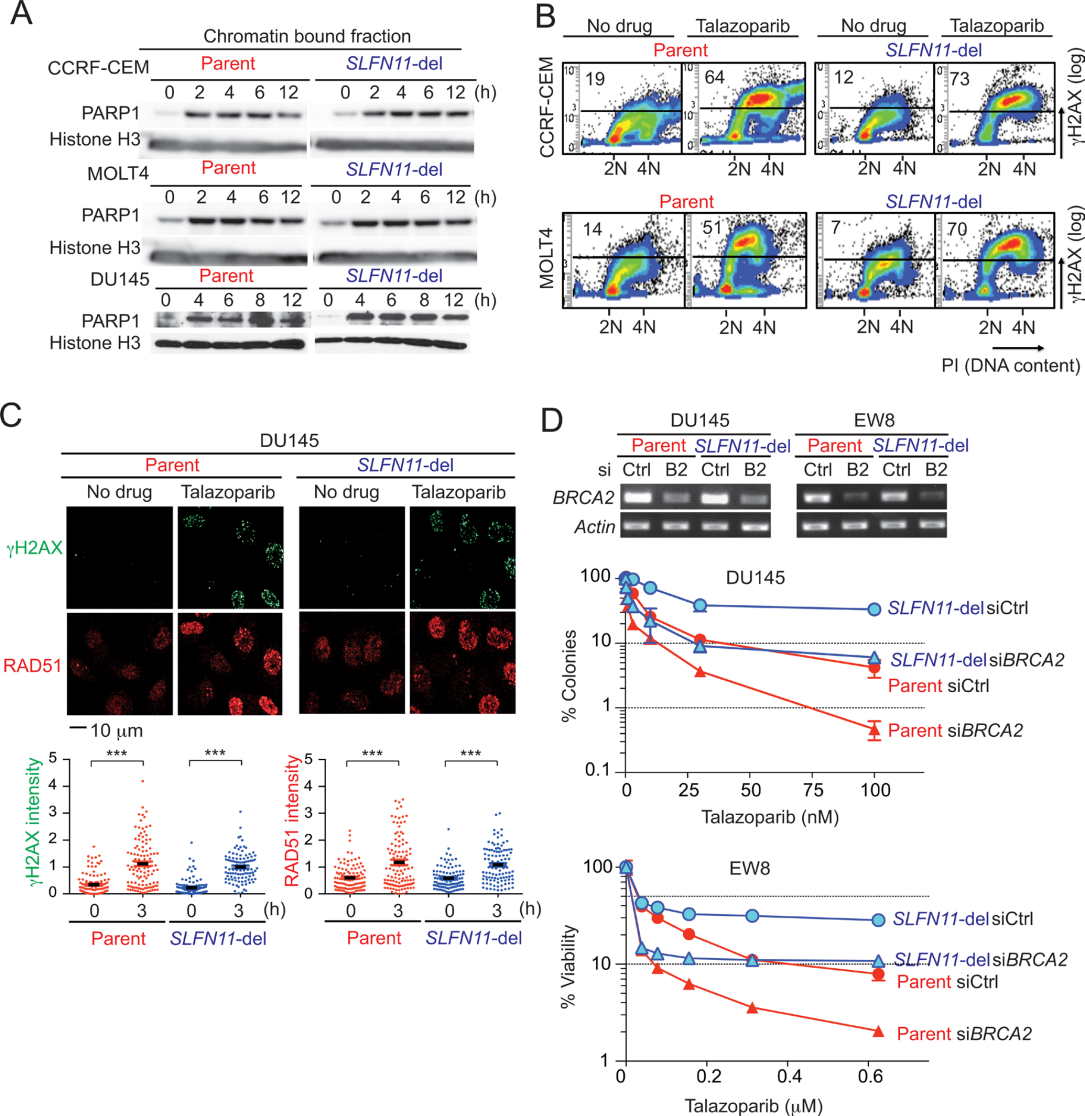
Comparable induction of DNA damage and homologous recombination regardless of *SLFN11* status **A.** PARP-DNA complexes were analyzed in parallel in parental (left) and *SLFN11*-del cells (right) in three cell line pairs (CCRF-CEM, MOLT4 and DU145) by Western blotting using chromatin-bound fractions. Cells were treated without drug (0) or with talazoparib (1 μM) plus methyl methanesulfonate MMS (0.001%) to enhance PARP trapping detection [8] for the indicated times. Blots were probed with the indicated antibodies. **B.** S-phase damage induction by talazoparib. The indicated cells were treated without or with talazoparib (1 μM) for 12 hours. γH2AX levels were analyzed by flow cytometry. DNA content stained with propidium iodide (PI) is on the x-axis and γH2AX levels measured by FITC signal the y-axis (logarithmic scale). The average population (%) of γH2AX-positive cells from 3 independent experiments is indicated at the top left in each panel. **C.** γH2AX and RAD51 foci formation in DU145 parental and *SLFN11*-del cells treated with or without talazoaprib (1 μM) for 3 hours. Representative confocal microscopy images are shown. Quantification was done with the ImageJ software (NIH). *N* = 99-115. ***p < 0.0001. **D.** BRCA2 functions in parallel with SLFN11. Transfection using control siRNA (siCtl) and *BRCA2* siRNA (si*B2*/ si*BRCA2*) was done in the indicated cell lines. The suppression of *BRCA2* mRNA was established by RT-PCR two days after transfection (top). Colony formation assay in DU145 cells (middle), and 72 hours viability assay in EW8 cells (bottom) were performed. Error bars represent SD (*n* ≥ 3)

Two well-established mechanisms of resistance to PARPIs include [33, 34]: 1/ reactivation of HR, which enables cells to overcome replicative damage [35-37], and 2/ activation of multidrug resistance (MDR) efflux pumps, which limits cellular drug levels [33]. To examine whether SLFN11 is involved in these mechanisms of resistance, first we checked the kinetics of PARP trapping in response to talazoparib [8] (Figure 3A). Similar accumulation of PARP1 in chromatin-bound fractions was observed regardless of the cellular *SLFN11* status, indicating that SLFN11 does not affect cellular penetration or efflux of talazoparib.

Next we examined replicative damage induced by PARP trapping and whether the effects of SLFN11 were related to HR. FACS analyses showed that cells in S- and G2-phase increased γH2AX level after talazoparib treatment regardless of *SLFN11* expression (Figure 3B), suggesting that replicative damage was induced by talazoparib regardless of the SLFN11 status. Intensity of γH2AX and RAD51 foci measured by immunofluorescence microscopy were also comparably increased in DU145 parental and *SLFN11*-del cells treated with talazoparib for 3 hours (Figure 3C). Because BRCA1/2 are necessary for the formation of RAD51 foci, the similar level of RAD51 foci formation in both cell lines indicates that the BRCA1/2 are functional in dependently of SLFN11.

To further examine the parallel activities SLFN11 and HR, we depleted *BRCA2* by siRNA transfection and compared the effects of BRCA2 inactivation in DU145 and EW8 parental and *SLFN11*-del cells (Figure 3D). Consistent with the known role of HR for PARPI response, BRCA2 depletion augmented the sensitivity to talazoparib in parental *SLFN11*-expressing DU145 and EW8 cells. Furthermore, BRCA2 depletion also reduced the viability of the *SLFN11*-del cells, and this sensitization was as extensive as in the case of the parental cells. These results demonstrate that HR is functional regardless of SLFN11, and that, SLFN11 is involved in a different pathway from the currently recognized HR and drug efflux pathways that determine response to PARPI.

### *SLFN11* induces prolonged S-phase arrest under talazoparib treatment, and exerts apoptosis

**Figure 4:**
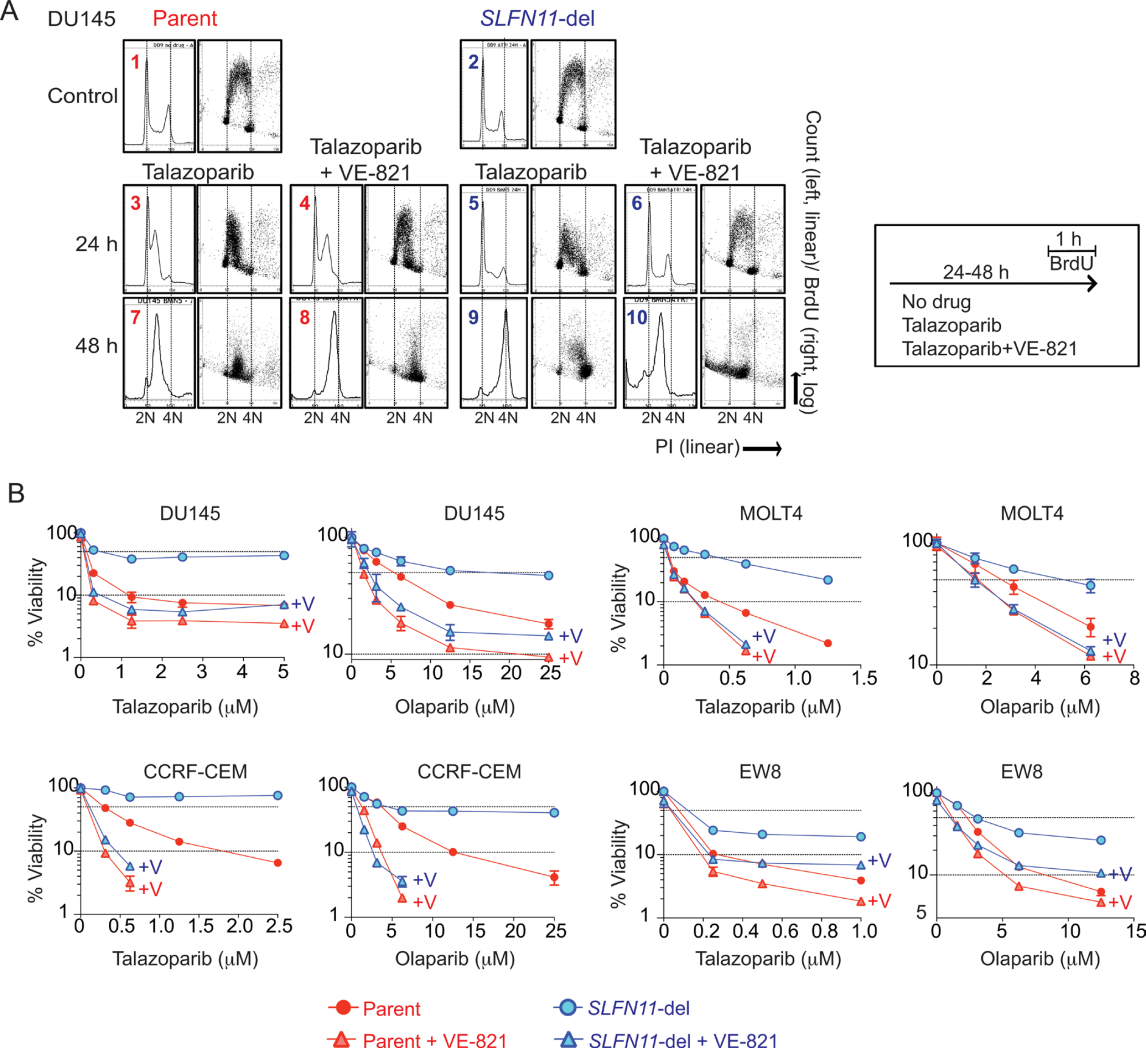
Enhanced activity of PARPIs by the ATR inhibitor (VE-821) in *SLFN11*-del cells **A.** Representative cell cycle analyses of DU145 parental and *SLFN11*-del cells treated as indicated for 24 or 48 hours. Experimental protocols are shown to the right. Vertical dashed lines correspond to 2N and 4N DNA contents. **B.** Cytotoxicity for the indicated cell lines in response to talazoparib and olaparib combined with or without 1 μM VE-821 (+V). Viability was normalized to untreated parental and *SLFN11*-del cells. Plots and experiments were done as in Figure 1C. Error bars represent SD (*n* = 3)

**Figure 5:**
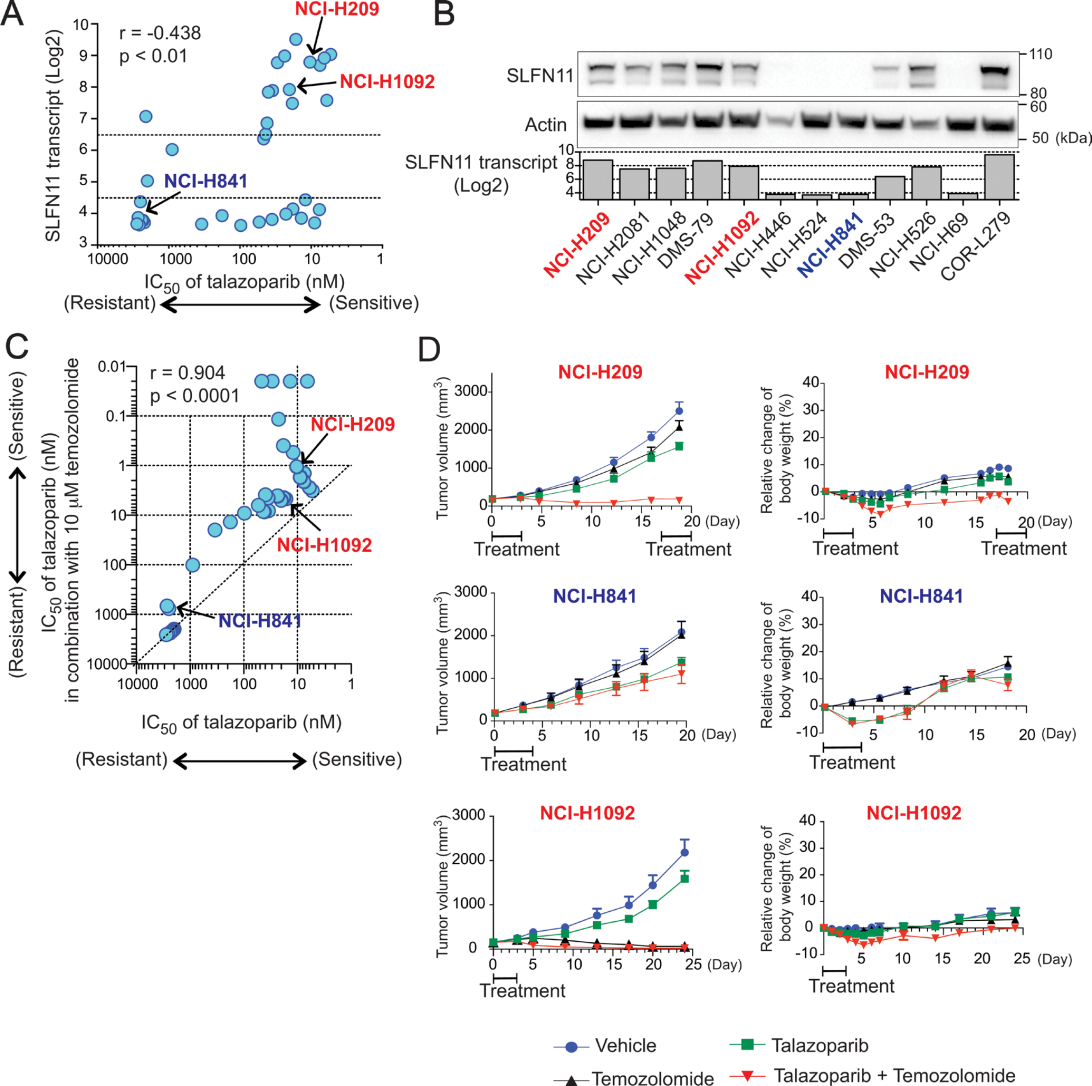
*SLFN11* expression is correlated with sensitivity to talazoparib as single-agent or combined with temozolomide in small cell lung cancer (SCLC) cells *in vitro* and *in vivo* **A.** Correlation between *SLFN11* expression (mRNA) and IC_50_ of talazoparib across SCLC cell lines. Pearson coefficient correlation: r=0.438, p<0.01. **B.** Selected SCLC cell lines were examined for *SLFN11* transcript and protein levels. Western blots of whole cell extract for the indicated cell lines and antibodies are compared to the *SLFN11* transcript level obtained from Broad CCLE database. **C.** Correlation between response to talazoparib in combination with 10 μM temozolomide (y-axis) and response to talazoparib as single agent (x-axis) across the SCLC cell lines. Pearson coefficient correlation: *r* = 0.9041, *p* < 0.0001. **D.** Mouse xenograft experiments using NCI-H209 (high *SLFN11*, high *MGMT*), NCI-H841 (low *SLFN11*, high *MGMT*) and NCI-H1092 (high *SLFN11*, low *MGMT*). Mice bearing tumor (volume ~125 mm^3^) were treated with vehicle, temozolomide, talazoparib, or the combination of both drugs. Treatment schedule is annotated in the graphs (see materials and methods). Tumor volume (left) and relative change of body weight (right) are plotted. Error bars represent SEM (*n* = 8)

Because talazoparib induces replicative DNA damage (Figure 3B), we examined the effects of SLFN11 on cell cycle progression after talazoparib treatment. In response to talazoparib, DU145 parental cells showed marked S-phase arrest with suppression of BrdU incorporation in mid- and late-S phase at 24 hours, and throughout S-phase at 48 hours (Figure 4A, 1, 3, 7). By contrast, the *SLFN11*-del cells showed an attenuated replication inhibition at 24 hours, and reached G2-phase (4N) at 48 hours (Figure 4A, 2, 5, 9). Similar results were obtained with the other three isogenic cell lines. They also showed higher G2 peaks in *SLFN11*-del cells than in the parental cells whereas the parental cells showed mid-S phase arrest under talazoparib treatment at 24 hours (Figure S3). Because prolonged replication fork stalling leads to lethal replisome disassembly and fork breakage [38], the prolonged S-phase arrest is likely to cause the hypersensitivity to PARP inhibitors in *SLFN11*-expressing cells.

Apoptosis analyses revealed that the percentage of apoptotic cells at 48 hours after talazoparib treatment increased from 9% to 29% and from 5% to 58% in DU145 and CCRF-CEM parental cells, respectively. By contrast, in the *SLFN11*-del DU145 and CCRF-CEM cells, the percentage of apoptotic cells increased only marginally from 5% to 9% and from 5% to 7%, respectively (Figure S4). These results imply that SLFN11 enforces S-phase arrest, and that prolonged S-phase arrest by SLFN11 induces apoptosis and causes hypersensitivity to PARP inhibitors.

### ATR inhibition overcomes the resistance of *SLFN11*-negative cells to PARPIs

Because ATR plays a major role in coordinating cell cycle progression and DNA repair in response to replicative damage, we examined whether SLFN11 affects the ATR-dependent S-phase checkpoint or not. We measured phospho-CHK1 (S345), a key effector of ATR [10, 11], after talazoparib treatment. Comparable levels phospho-CHK1 (S345) were observed in parental and *SLFN11*-del cells across the four cell line pairs (Figure S5), demonstrating that ATR activation by talazoparib is independent of SLFN11.

**Figure 6:**
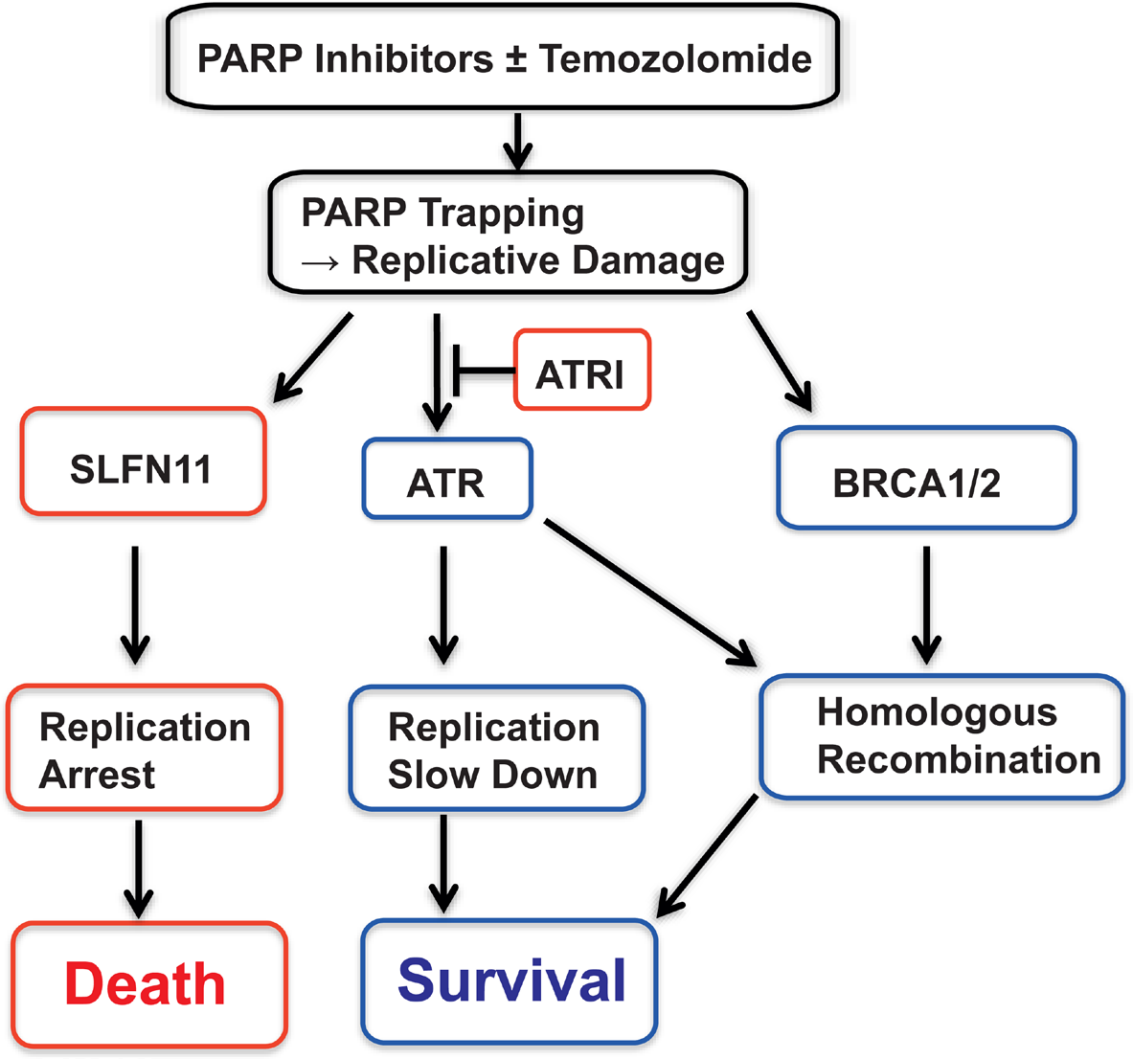
Summary scheme proposing the role of SLFN11 in parallel to ATR and homologous recombination (BRCA1/2) Red boxes indicate disadvantage factors for cell survival, while blue boxes indicate supportive factors for cell survival. See Discussion for details.

To determine whether the SLFN11-dependent S-phase arrest was linked to ATR, we combined the ATR inhibitor (VE-821) with talazoparib. Addition of the ATR inhibitor recovered replication almost completely at 24 hours in *SLFN11*-del cells, but induced incomplete replication with accumulation of sub-G1 population at 48 hours (Figure 4A, 6 and 10), indicating that ATR-mediated S-phase checkpoint dominantly regulates cell cycle progression in the *SLFN11*-del cells. On the other hand, the effect of ATR inhibition was marginal in the parental cells at 24 and 48 hours with the bulk of the S-phase peak only slightly shifted to 4N (Figure 4A, 4 and 8). These results show that SLFN11 inhibits DNA replication in parallel to ATR-mediated S-phase checkpoint.

ATR inhibitors, which are in clinical development [17], synergize with DNA damaging agents including topoisomerase inhibitors, gemcitabine, cisplatin and veliparib by abrogating the S-phase checkpoint, resulting in DNA damage accumulation. Because ATR inhibition had marginal impact on cell cycle in *SLFN11*-positive cells, while it had substantial impact in *SLFN11*-negative cells, we examined whether the ATR inhibitor had a different impact on the viability of *SLFN11*-positive and -negative cells. Viability assays with PARP inhibitors combined with or without VE-821 showed that ATR inhibition was synergistic both with talazoparib and olaparib in both the parental and *SLFN11*-del cells (Figure 4B). However, the synergy was consistently greater (with lower Combination Index (CI) values) for all four isogenic cell lines tested in the *SLFN11*-del than in the parental cells (Figure 4B and Table S1).

Consistent to the results of cell cycle and viability assays, apoptotic cells increased from 9% to 34% in *SLFN11*-del DU145 cells, while they were already high (29%) with talazoparib alone, and increased only slightly (from 29% to 38%) by ATR inhibition in *SLFN11*-positive DU145 cells (Figure S4A). Similar results were obtained using CCRF-CEM parent and *SLFN11*-del cells (Figure S4B). These results demonstrate the potential value of combining talazoparib or olaparib with ATR inhibitors, especially as a way to overcome the resistance of *SLFN11*-negative cells to PARP inhibitors.

### Screening with 36 small cell lung cancer (SCLC) cell lines reveals significant correlation between *SLFN11* expression and talazoparib sensitivity

Because of the promising activity of PARP inhibitors for patients with SCLC [39, 40], we extended our findings from the NCI-60 and our four isogenic *SLFN11*-del cell lines to a panel of 36 SCLC cell lines (Table S2). All cells have published genomic profiles (gene expression data) from the Broad CCLE portal site, and examination of *SLFN11* expression revealed a non-normal distribution with half of the cell lines (18 out 36) having minimal or no *SLFN11* expression (Log2 value below 4.5) and 15/36 having high *SLFN11* expression (Log2 value above 6.5) (Figure 5A, Y-axis). Next, we measured the IC_50_ of talazoparib (50% inhibition concentration) across the 36 SCLC cell lines (Table S3). *SLFN11* transcript levels were significantly correlated to the IC_50_ of talazoparib (*p* < 0.01, Figure 5A). We also measured SLFN11 protein levels in the SCLC lines by Western blotting and found that protein levels matched *SLFN11* transcripts (Figure 5B), which is consistent with our NCI-60 data (Figure 1B) [23].

Because talazoparib in combination with temozolomide results in remarkable synergy [29] and activity in Ewing's sarcoma models [41], we evaluated the combination of temozolomide with talazoparib across our SCLC cell line panel. We combined a non-toxic dose of temozolomide (10 μM) with a range of talazoparib concentrations, and determined the IC_50_ of talazoparib in the presence and absence of temozolomide (Table S3). The activity of talazoparib alone was highly correlated with the activity of the combination talazoparib-temozolomide across the 36 cell lines (Pearson's *r* = 0.904, *p* < 0.0001, Figure 5C), supporting the notion that talazoparib is the cytotoxic component of the combination [29]. Moreover, temozolomide reduced the IC_50_ values of talazoparib by a factor of approximately 10-fold (Figure 5C). Collectively, these data demonstrate that, in SCLC cell lines, *SLFN11* expression determines cellular sensitivity to both talazoparib and the talazoparib-temozolomide combination, suggesting the potential value of combining temozolomide with talazoparib in *SLFN11*-positive SCLC.

### The combination talazoparib-temozolomide shows greater synergy in SCLC xenograft models with *SLFN11*-positive than *SLFN11*-negative cells

Next we examined the talazoparib-temozolomide combination in xenograft models using three SCLC cell lines harboring different *SLFN11* and *MGMT* status: NCI-H209 (high *SLFN11*, high *MGMT*), NCI-H841 (low *SLFN11*, high *MGMT*) and NCI-H1092 (high *SLFN11*, low *MGMT*) (Table S3) (Figure 5D). A preceding paper showed that MGMT-positive cancer cells strongly respond to the combination of temozolomide and PARP inhibitors (PARPi), whereas MGMT-deficient cells do not because MGMT-negative cells are primarily killed by unrepaired O6-methyl-guanine generated by low dose of temozolomide [42].

As NCI-H209 cells (with high *SLFN11* expression) respond well to daily talazoparib treatment [43], in this study, to examine the effect of talazoparib + temozolomide combination, we administrated drugs during the initial 4 or 5 days, and then left mice without drugs for at least 14 days. We first tested a range of combination regimen using low doses of temozolomide, and found that temozolomide at 3 mg/kg/day for 4-5 days plus talazoparib at 0.25-0.33 mg/kg/day for 4-5 days can be tolerated in different host mouse strains (Figure 5D). We then conducted comparative assessment of each single agent versus the combination of talazoparib plus temozolomide in these models. Although temolozomide and talazoparib alone had marginal anti-tumor activity in NCI-H209 and NCI-H841 xenografts models, the combination induced marked synergy in NCI-H209 (high *SLFN11*) but not in NCI-H841 (low *SLFN11*) (Figure 5D). On the other hand, temozolomide alone was sufficient to reduce tumor growth in NCI-H1092 (low *MGMT*) xenograft (Figure 5D). These results show that *SLFN11* expression can determines cellular sensitivity to talazoparib and temozolomide combination treatment in tumor models, and that temozolomide single treatment can be sufficient to reduce tumor growth if tumor cells are MGMT-negative.

## DISCUSSION

Our study establishes SLFN11 as a causal and dominant determinant of cellular response to PARPIs (talazoparib and olaparib), given as single agents and in combination with temozolomide. It is the first report demonstrating that SLFN11 affect cellular responses to PARPIs independently of homologous recombination and drug efflux by blocking DNA replication independently of ATR. Moreover, we provide the rationale and proof of concept for evaluating SLFN11 as a potentially important biomarker of response for patients treated with PARPIs, and for combining ATR and PARP inhibitors to overcome the resistance of *SLFN11*-negative cells to PARPIs.

### Clinical implications of *SLFN11* for precision medicine

A notable consideration is that the impact of SLFN11 is dependent on PARP trapping, which depends on the PARPI (talazoparib >> niraparib ≈ olaparib ≈ rucaparib >> veliparib) [8, 9]. Our data mining of the NCI-60 readily picked talazoparib, the most potent PARP trapping inhibitor [7] as showing a highly significant correlation between cancer cell killing and *SLFN11* transcript levels (Figure 1A). We also found that olaparib sensitivity but not veliparib sensitivity was linked to *SLFN11* expression (Figure 1C). Moreover, highly significant correlation between *SLFN11* expression and olaparib response also emerges from the recently released independent database including 780 individual cancer cell lines treated with olaparib (http://www.broadinstitute.org/ctrp/) [44]. Yet, the impact of SLFN11 status on drug sensitivity was higher for talazoparib than for olaparib throughout our experiments. Consistent with the connection between PARP-trapping and *SLFN11*, we found that increasing PARP-trapping by combining talazoparib or olaparib with temozolomide [29] yielded *SLFN11*-dependent response (Figure 2B). Although veliparib has weak PARP-trapping potency [8, 9], it can exert cytotoxicity with temozolomide by catalytic inhibition and by PARP-trapping at high dose [45]. As our study went to press, similar conclusions were reported by Lok et al. using SCLC cell lines and SCLC patient derived xenograft models [46]. Together both studies imply the potential of *SLFN11* expression as a dominant biomarker to predict response to PARPIs as single agent acting by trapping PARP and damaging DNA (talazoparib, olaparib, and probably niraparib and rucaparib), as well as for combination regimens of broad PARPis with temozolomide, which are in a large number of ongoing clinical trials.

The synthetic lethality of PARPIs for BRCA-deficient cells is an elegant strategy, but the reality is that not all BRCA deficient tumors respond to PARP inhibitors [47, 48], and that PARPIs are also active beyond homologous recombination deficiencies (HRD) [8, 49, 50]. Here we show that *SLFN11* expression sensitizes to PARPIs in parallel to BRCA-deficiency status (Figures 3 and 6). Therefore, tumors harboring HRD as well as *SLFN11* expression should be better responders than tumors harboring either parameter. By contrast, tumors without HRD and lacking *SLFN11* expression should be the worst responder for PARPIs (Figure 3D). Because approximately 45% of cancer cell lines are *SLFN11*-negative at the transcript level [23, 25], frequently by promoter hypermethylation [24], SLFN11 can be considered a testable biomarker to predict responders to PARPIs in addition to BRCA mutations and HRD. SLFN11 is detectable by immunohistochemistry from tissue samples [27] [46]. Because SLFN11 determines response to a wide range of DNA damaging drugs in addition to PARPIs, further clinical studies and assays are warranted to score SLFN11 expression in tumor samples like estrogen receptor is examined routinely by immunohistochemistry in breast cancer.

### How does *SLFN11* sensitize cancer cells to PARPIs?

Two well-established mechanisms of resistance to PARPIs include [33, 34]: 1/ reactivation of HR, which enables cells to overcome replicative damage [35-37], and 2/ activation of multidrug resistance (MDR) drug efflux pumps, which limits cellular drug levels [33]. However, neither is related to SLFN11, as SLFN11 does not affect DNA damage level at early time point (PARP-trapping, γH2AX and RAD51 level, Figure 3A and 3C). We conclude that HR is functional regardless of *SLFN11* expression, which seems contradictory to the recent publication of Mu et al. [28], who found that SLFN11 inhibits checkpoint maintenance and homologous recombination by removing RPA on single stranded DNA. However, their conclusion was based on data collected at 24 and 48 hours after camptothecin pulse treatment (1 hour treatment, and then wash and release in drug free medium) when cell cycle distributions are different between *SLFN11*-positive and negative cells [23]. Our study shows that SLFN11 induces prolonged S-phase arrest at least until 48 hours after continuous talazoaparib treatment while *SLFN11*-negative cells continue cell cycle progression until reaching G2-phase (Figure 4A). Because sister chromatids are not fully available under the condition where replication is blocked at mid-S-phase by SLFN11, it is plausible that, at relatively late time points, SLFN11 indirectly reduces HR marked by RPA and RAD51 foci, and reduces ATR activation due to diminished RPA loading. Consistent to the report by Mu et al., we observed significantly higher RAD51 foci formation in *SLFN11*-del cells than *SLFN11*-positive cells at 24 hours after talazoparib treatment (data not shown). We do not exclude the possibility that SLFN11 inhibits HR via removal of RPA polymer as proposed by Mu et al. [28]. However, we and Mu et al. observed comparable RAD51 foci formation regardless of SLFN11 at early time points after drug treatment (Figure 3C), indicating that BRCAs are properly working for RAD51 deposition, and that SLFN11 does not directly interfere with HR factors like BRCAs. Our experiments using siRNA BRCA2 support our conclusion that SLFN11 acts in parallel with HR (Figures 3 and 6). Hence, we conclude that resistance in *SLFN11*-deficient cells is caused neither by impaired drug penetration nor by activation of homologous recombination but by sustained cellular replicative potential following DNA damage.

Our data clearly show that SLFN11 inhibits replication and forces cell cycle arrest at mid S-phase under talazoparib treatment, while S*LFN11*-negative cells keep replicating and reach G2 (Figure 4A). Because prolonged stalling of replication forks lead to lethal replisome disassembly and fork breakage [38], the prolonged S-phase arrest by SLFN11 is likely the cause of *SLFN11*-dependent cell killing by PARP inhibitors. Indeed, we found that apoptotic cell populations increased after talazoparib treatment in *SLFN11*-positive cells (Figure S5). Hence, we propose that prolonged S-phase arrest by SLFN11 exerts apoptosis and hypersensitivity to PARP inhibitors. Further studies are warranted to elucidate the molecular details of how SLFN11 inhibits replication.

### Rationale for combining ATR and PARP inhibitors to overcome resistance to PARPIs due to *SLFN11* inactivation

Although lack of *SLFN11* expression is a major cause of resistance to PARP inhibitors, we demonstrate that the addition of an ATR inhibitor overcomes such resistance (Figure 4). ATR is a guardian for S-phase cell cycle under replicative damage [10, 11]. ATR inhibition promotes unscheduled origin firing, and generates excess single strand DNA leading to fork breakage and cell death [16]. While ATR inhibition kills cell by accelerating replication under replicative stress, in an opposite way, SLFN11 kills cells by enforcing prolonged S-phase arrest under PARP inhibitor treatment. Our experiments show that ATR and PARP inhibitor combination synergizes more in *SLFN11*-del cells than *SLFN11*-positive cells (Figure 4B and Table S1). Figure 6 provides a schematic representation of the role of SLFN11 in the context of ATR activation. In response to replicative damage by PARP trapping, *SLFN11*-positive cells use dual cell cycle regulation: one is *SLFN11*-dependent prolonged replication arrest leading to cell death, and the other is ATR-dependent S-phase checkpoint that slows down cell cycle and promotes cell survival. By contrast, *SLFN11* deficient cells rely primarily on ATR activation for their cell cycle regulation under the replicative damage. This creates a synthetic lethality scenario [49, 50] for ATR inhibitors in *SLFN11*-deficient cells as the combination of PARP and ATR inhibitors abolishes cell cycle regulation completely in *SLFN11*-deficient cell, but only partially in the parental cells. Thus, the ATR-PARP inhibitors combination has more impact in *SLFN11*-negative than in *SLFN11*-positive cells. This conclusion could have broad implications as 45-50% of cancer cell lines inactivate *SLFN11* [23-25].

In addition, our findings provide a link between the marked sensitivity of Ewing's sarcoma (EWS) cells to olaparib [51] and the high *SLFN11* expression in EWS cells [25]. Combined with our recent finding that FLI1, a transcription factor, upregulates of *SLFN11* expression [26], the link between EWS cells and the hypersensitivity to PARP inhibitors can be derived from the high *SLFN11* expression induced by *EWS-FLI1* translocations in EWS cells. An additional mechanism of hypersensitivity of EWS cells to PARP inhibitors could be the importance of PARP1 as a cofactor of FLI1 based on protein-protein interaction between EWS-FLI1 and PARP1, and EWS-FLI1:PARP1-positive feedback loop in transcriptional activation [52]. Because, Ewing's sarcoma initially responds to DNA damaging agents, for which cell killing depends on SLFN11 [22, 24, 44], it will be important to determine the SLFN11 status of tumors in patients at relapse.

In summary, our study reveals the relevance of SLFN11 for PARPIs given alone and in combination with temozolomide. Developing assays for assessing SLFN11 status as a predictive marker for tumor response to DNA damaging agents and clarifying the molecular details underlying SLFN11 biology are pressing tasks for the future.

## EXPERIMENTAL PROCEDURES

### Cell line, culture and drugs

DU145, CCRF-CEM, MOLT4, and K562 were obtained from the Division of Cancer Treatment (DCTD), Developmental Therapeutics Program (DTP, NCI), and EW8 and A673 are kind gifts from Dr. Lee Helman (NCI/NIH). All cells were grown in RPMI medium with 10% FBS (Gibco-BRL) at 37°C in 5% CO_2_. Information about the SCLC lines is shown in Table S2. The ATR inhibitor VE-821, olaparib, and veliparib were obtained from the DCTD. Talazoparib was provided by BioMarin Pharmaceutical Inc. Temozolomide (T2577) and methyl methanesulfonate MMS (129925) were purchased from Sigma-Aldrich.

### Drug cytotoxicity data of the NCI-60

The cell viability assays across the NCI-60 cell panel were obtained from the DTP, NCI (https://dtp.cancer.gov/discovery_development/nci-60/default.htm) [53, 54]. Further details can be found at the CellMiner website [20] (https://discover.nci.nih.gov/cellminer/).

### Cell viability assays

Cells were continuously exposed to the indicated drug concentrations for 72 hours in triplicate. Five thousand cells for CCRF-CEM, MOLT4 and K562, and 1,500 cells for SF295, DU145, EW8, A673, MDA_MB231, H29 and HCT116 cells were seeded in 96-well white plates (#6005680 Perkin Elmer Life Sciences) in 100 μl of medium per well. Cellular viability was determined using ATPlite 1-step kits (PerkinElmer). The ATP level in untreated cells was defined as 100%. Viability of treated cells was defined as: (ATP in treated cells)/(ATP in untreated cells)x 100. The 36 SCLC cell lines (obtained from American Type Culture Collection, European Collection of Cell Cultures or Japanese Collection of Research Bioresources, Table S2) were grown in vendor-suggested media and seeded in 96 well plates at pre-determined cell density based on cell doubling time. After 24 hours, talazoparib at 2000, 400, 80, 16, 3.2, 0.64 nM in 0.2% DMSO was added in duplicate, and incubated for additional 5 or 7 days. Cell viability was determined by CellTiter Glo assay (Promega). IC_50_ (inhibition concentration 50%) was calculated by the treated cell counts relative to untreated control using GraphPad Prism5.

### Clonogenic assays

Treated or untreated cells were plated onto six-well plates and incubated with or without drug-containing medium continuously for 10 days to allow colony formation. Colonies were then fixed and stained with 0.05% (wt/vol) methylene blue (Sigma-Aldrich).

### Immunoblotting

To prepare whole cell lysates, cells were lysed with the CelLytic™M lysis reagent (C2978, Sigma-Aldrich). After thorough mixing and incubation at 4°C for 30 min, lysates were centrifuged at 15,000 g at 4°C for 10 min, and supernatants were collected. To prepare chromatin-bound subcellular fractions, we followed the protocol of Subcellular Protein Fractionation Kit from Thermo Scientific (78840) [8]. Immunoblotting was carried out using standard procedures.

### Analyses of cell cycle and apoptosis

Cells were incubated with 10 μM 5-bromo-2′-deoxyuridine (BrdU) for 1 hour before fixation with 70% ethanol. BrdU was detected by flow cytometry (anti-BrdU FITC, BD Biosciences, 347583 following the manufacturer protocol). Apoptotic cells were detected 48 hours after talazoparib treatment using Annexin V/PI costaining (FITC Annexin V Apoptosis kit; BD Biosciences). Propidium iodide (PI) was used to measure DNA content. Cells were analyzed on a FACScan flow cytometer (Becton Dickinson).

### Immunofluorescence microscopy

Cells were fixed for 10 min with freshly prepared 4% (wt/vol) paraformaldehyde in phosphate buffered saline (PBS), and then permeabilized for 10 min in 0.5% Triton-X100/PBS. Samples were then blocked with 5% bovine serum albumin/0.1% Tween 20/PBS for 1 hour, and incubated for 2 hours with primary antibodies. After washing with 0.1% Tween 20/PBS, cells were stained with Alexa Fluor 488 and/or Alexa Fluor 568 for 1 hour. Images were captured with a confocal microscope (Nikon PCM2000). Quantification of signal intensity for γH2AX and RAD51 was done using the ImageJ software. Circles slightly smaller than a regular nucleus size was set to quantify signals in individual nuclei for each image. Circle of same size was used throughout the measurement. Mean intensity of both signals (γH2AX and RAD51) was displayed as scatter plots to compare cell lines and conditions.

### Generation of *SLFN11*-deleted cells

To delete the *SLFN11* gene, we designed two independent guide RNAs (A and B) targeting just downstream of the start codon in the 4^th^ exon using the CRISPR design tool (http://crispr.mit.edu) [55]. A human codon-optimized SpCas9 and chimeric guide RNA expression plasmid (pX330: pX330-U6-chimeric_BB-CBh-hSpCas9) was purchased from Addgene. Each guide RNA was inserted into the pX330 plasmid (pX330-A, and pX330-B). The gene-targeting constructs harboring homology arms and a puromycin-resistance gene were prepared. Briefly, ~1 kb genomic sequences just upstream and downstream of the Cas9 cleavage sites were amplified by PCR methods from genomic DNA. The PCR products of upstream site (left homology arm) and downstream site (right homology arm) were subcloned into pCR2.1-TOPO vector (Invitrogen) at TA cloning site and ApaI/XhoI restriction endonuclease site, respectively in the desired direction. Puromycin resistance gene was finally subcloned between the homology arms at the NotI restriction endonuclease site. The targeting construct and pX330-A or pX330-B were co-transfected into DU145 and EW8 cells by lipofection and into CCRF-CEM and MOLT cells by electroporation. After transfection, cells were released into drug-free medium for 48 hours followed by puromycin selection until single colonies were formed. Single clones were expanded, and gene-deletion was confirmed by Western blotting. PCR primers and guide RNA sequences will be provided on request.

### Generation of *SLFN11*-expressing cells

*SLFN11* cDNA was amplified using the forward primer (5′- ATCGGATCC GCGGCCAACATGGAGGCAAATCAGTGC-3′) and the reverse primer with the sequence for the Flag tag (5′-ATTGTCGACGCGGCCCTACTTATCGT CGTCAT CCTTGTAATCATGGCCACCCCACGGAA-3′) and cloned into pCDH-EF1-MCS-(PGK-copGFP) lentiviral expression vector (System Biosciences) by In-Fusion HD cloning kit (Clontech). The lentiviral *SLFN11*-expressing vector and the pPACKH1 lentivector packaging plasmids were cotransfected into 293TN cells (System Biosciences) and the viral particles were collected to infect K562 cells with Transdux™ (System Biosciences). The *SLFN11*-expressing cells with GFP signal were sorted using a Fluorescence Activated Cell Sorter (FACS).

### Antibodies

Antibodies against PARP1 (sc-7150), CHK1 (sc-8408), RAD51 (sc-8394) and SLFN11 (E-4) (sc-374339) were obtained from Santa Cruz; phospho-CHK1 (S345) (ab58567) and GAPDH (ab9485) from Abcam; γH2AX (05-636) and histone H3 (07-690) from Upstate Biotechnology; and actin (A3853) from Sigma-Aldrich. Secondary antibodies were horseradish peroxidase (HRP)-conjugated antibodies to mouse or rabbit IgG (GE Healthcare, UK).

### siRNA transfection and RT-PCR

Gene-specific siRNAs (mix of four sequences) for human BRCA2 (L-003462-00-0005), human PARP1 (L-006656-00-0005), human SLFN11 (L-016764-01-0005) and negative control siRNA (D-001810-10) were products of Dharmacon (Lafayette, CO, USA). Ten nanomolar of each siRNA was transfected to DU145 or EW8 cells with Lipofectamin RNAiMAX Reagent (13778, Invitrogen) according to the manufacturer's instructions. Culture medium was changed 6-8 hours after the transfection. Two days after the transfection, total RNA was extracted using TRIzol reagent (Invitrogen), followed by further purification using PureLink RNA Mini Kit (Life Technology) with DNase treatment (Qiagen). Complementary DNA (cDNA) was synthesized using SuperScript II Reverse Transcriptase Kit (Invitrogen). To amplify human BRCA2 cDNA, forward primer 5′-GAGGCCTGTAAAGACCTTGAATTA-3′, and reverse primer 5′-GATTTGTGTAACAAGTTGCAGGAC-3′ were used.

### Microarray data of CCLE

The genome-wide gene expression data of CCLE (http://www.broadinstitute.org/ccle/home) cell lines were available online. Gene expression profiles of small cell lung cancer samples used in this study were downloaded and extracted from the CCLE data portal above (file CCLE_Expression_Entrez_2012-09-29.gct).

### Xenograft experiments

Female Balb/c nude mice (6-8 week old) were obtained from Shanghai BK Laboratory Animal Center (Shanghai, China), and female NCr *nu/nu* mice were purchased from Charles River Laboratories, Inc. (Frederick, MD). All the procedures related to animal handling, care and the treatment in this study were approved by the Institutional Animal Care and Use Committee (IACUC) of Shanghai Chempartner or IACUC of Southern Research, in accordance with the regulations of the Association for Assessment and Accreditation of Laboratory Animal Care (AAALAC). For talazoparib and temozolomide combination experiments, NCI-H209 or NCI1092 tumor cells were injected s.c. in the flank of CB17 female SCID mice (Beijing Vital River Laboratory Animal Co., Ltd, Beijing, China), NCI-H841 tumor cells were infected s.c. in the flank of Balb/c female nude mice. When tumors reached ~150 mm^3^ average volume, animals were randomized into treatment groups (*n* = 6-8 per group). NCI-H209 and NCI-H1092 tumors were treated by oral gavage once daily on days 1-4 and 17-20 with either vehicles, talazoparib (0.25 mg/kg), temozolomide (3 mg/kg), or in combination in the same dose and schedule as corresponding single agent; NCI-H841 tumors were treated by oral gavage on days 1-5 with either vehicles, talazoparib (0.165 mg/kg) twice daily, temozolomide (3 mg/kg) once daily, or in combination (talazoparib 0.165 twice daily, temozolomide 3 mg/kg once daily).

### Statistical analyses

Correlations of gene expression and drug cytotoxicity were performed using the Pearson's correlation coefficient and considered significant with an uncorrected two-tailed *p* < 0.05. The two-tailed independent samples t-test was applied to determine the statistical significance of the differences between the two experimental groups. Such analyses were carried out using GraphPad Prism version 5.0 (GraphPad Software, http://www.graphpad.com).

### Analysis of combination effects

The synergism analysis for the combination effects was analyzed using the Chou-Talalay method [56]. Combination Index (CI) of each combination treatment was calculated using CalcuSyn software (Biosoft, Inc., Cambridge, United Kingdom), and CI: 0.3-0.7, CI: 0.1-0.3 and CI: < 0.1 were defined as synergism, strong synergism and very strong synergism, respectively [57].

## SUPPLEMENTARY MATERIALS FIGURES AND TABLES



## References

[1] Rouleau M, Patel A, Hendzel MJ, Kaufmann SH, Poirier GG (2010). PARP inhibition: PARP1 and beyond. Nat Rev Cancer.

[2] Shen Y, Rehman FL, Feng Y, Boshuizen J, Bajrami I, Elliott R, Wang B, Lord CJ, Post LE, Ashworth A (2013). BMN 673, a novel and highly potent PARP1/2 inhibitor for the treatment of human cancers with DNA repair deficiency. Clin Cancer Res.

[3] Benjamin RC, Gill DM (1980). ADP-ribosylation in mammalian cell ghosts. Dependence of poly(ADP-ribose) synthesis on strand breakage in DNA. J Biol Chem.

[4] Durkacz BW, Omidiji O, Gray DA, Shall S (1980). (ADP-ribose)n participates in DNA excision repair. Nature.

[5] Bryant HE, Schultz N, Thomas HD, Parker KM, Flower D, Lopez E, Kyle S, Meuth M, Curtin NJ, Helleday T (2005). Specific killing of BRCA2-deficient tumours with inhibitors of poly(ADP-ribose) polymerase. Nature.

[6] Farmer H, McCabe N, Lord CJ, Tutt AN, Johnson DA, Richardson TB, Santarosa M, Dillon KJ, Hickson I, Knights C, Martin NM, Jackson SP, Smith GC (2005). Targeting the DNA repair defect in BRCA mutant cells as a therapeutic strategy. Nature.

[7] Murai J, Huang SY, Renaud A, Zhang Y, Ji J, Takeda S, Morris J, Teicher B, Doroshow JH, Pommier Y (2014). Stereospecific PARP trapping by BMN 673 and comparison with olaparib and rucaparib. Mol Cancer Ther.

[8] Murai J, Huang SY, Das BB, Renaud A, Zhang Y, Doroshow JH, Ji J, Takeda S, Pommier Y (2012). Trapping of PARP1 and PARP2 by Clinical PARP Inhibitors. Cancer Res.

[9] Murai J, Pommier Y, Sharma NJCaRA (2015). Classification of PARP inhibitors based on PAPR trapping and catalytic inhibition, and rationale for combination with topoisomerase I inhibitors and alkylating agents. PARP inhibitors for Cancer Therapy.

[10] Nam EA, Cortez D (2011). ATR signalling: more than meeting at the fork. Biochem J.

[11] Zeman MK, Cimprich KA (2014). Causes and consequences of replication stress. Nat Cell Biol.

[12] Friedel AM, Pike BL, Gasser SM (2009). ATR/Mec1: coordinating fork stability and repair. Curr Opin Cell Biol.

[13] Seiler JA, Conti C, Syed A, Aladjem MI, Pommier Y (2007). The Intra-S-Phase Checkpoint Affects both DNA Replication Initiation and Elongation: Single-Cell and -DNA Fiber Analyses. Mol Cell Biol.

[14] Cliby WA, Roberts CJ, Cimprich KA, Stringer CM, Lamb JR, Schreiber SL, Friend SH (1998). Overexpression of a kinase-inactive ATR protein causes sensitivity to DNA-damaging agents and defects in cell cycle checkpoints. EMBO J.

[15] Claus Storgaard Sørensen HB, Viola Nähse-Kumpf, Randi G, Seligmann DH (2011). Syljuåsen. Faithful DNA Replication Requires Regulation of CDK Activity by Checkpoint Kinases.

[16] Josse R, Martin SE, Guha R, Ormanoglu P, Pfister TD, Reaper PM, Barnes CS, Jones J, Charlton P, Pollard JR, Morris J, Doroshow JH, Pommier Y (2014). ATR inhibitors VE-821 and VX-970 sensitize cancer cells to topoisomerase i inhibitors by disabling DNA replication initiation and fork elongation responses. Cancer Res.

[17] Karnitz LM, Zou L (2015). Molecular Pathways: Targeting ATR in Cancer Therapy. Clin Cancer Res.

[18] Shoemaker RH (2006). The NCI60 human tumour cell line anticancer drug screen. Nat Rev Cancer.

[19] Holbeck SL, Collins JM, Doroshow JH (2010). Analysis of Food and Drug Administration-approved anticancer agents in the NCI60 panel of human tumor cell lines. Mol Cancer Ther.

[20] Reinhold WC, Sunshine M, Varma S, Doroshow JH, Pommier Y (2015). Using CellMiner 1. 6 for Systems Pharmacology and Genomic Analysis of the NCI-60. Clin Cancer Res.

[21] Zeeberg BR, Reinhold W, Snajder R, Thallinger GG, Weinstein JN, Kohn KW, Pommier Y (2012). Functional categories associated with clusters of genes that are co-expressed across the NCI-60 cancer cell lines. PLoS One.

[22] Sousa FG, Matuo R, Tang SW, Rajapakse VN, Luna A, Sander C, Varma S, Simon PH, Doroshow JH, Reinhold WC, Pommier Y (2015). Alterations of DNA repair genes in the NCI-60 cell lines and their predictive value for anticancer drug activity. DNA Repair (Amst).

[23] Zoppoli G, Regairaz M, Leo E, Reinhold WC, Varma S, Ballestrero A, Doroshow JH, Pommier Y (2012). Putative DNA/RNA helicase Schlafen-11 (SLFN11) sensitizes cancer cells to DNA-damaging agents. Proc Natl Acad Sci U S A.

[24] Nogales V, Reinhold WC, Varma S, Martinez-Cardus A, Moutinho C, Moran S, Heyn H, Sebio A, Barnadas A, Pommier Y, Esteller M (2016). Epigenetic inactivation of the putative DNA/RNA helicase SLFN11 in human cancer confers resistance to platinum drugs. Oncotarget.

[25] Barretina J, Caponigro G, Stransky N, Venkatesan K, Margolin AA, Kim S, Wilson CJ, Lehar J, Kryukov GV, Sonkin D, Reddy A, Liu M, Murray L (2012). The Cancer Cell Line Encyclopedia enables predictive modelling of anticancer drug sensitivity. Nature.

[26] Tang SW, Bilke S, Cao L, Murai J, Sousa FG, Yamade M, Rajapakse V, Varma S, Helman LJ, Khan J, Meltzer PS, Pommier Y (2015). SLFN11 Is a Transcriptional Target of EWS-FLI1 and a Determinant of Drug Response in Ewing Sarcoma. Clin Cancer Res.

[27] Deng Y, Cai Y, Huang Y, Yang Z, Bai Y, Liu Y, Deng X, Wang J (2015). High SLFN11 expression predicts better survival for patients with KRAS exon 2 wild type colorectal cancer after treated with adjuvant oxaliplatin-based treatment. BMC Cancer.

[28] Mu Y, Lou J, Srivastava M, Zhao B, Feng XH, Liu T, Chen J, Huang J (2016). SLFN11 inhibits checkpoint maintenance and homologous recombination repair. EMBO Rep.

[29] Murai J, Zhang Y, Morris J, Ji J, Takeda S, Doroshow JH, Pommier YG (2014). Rationale for PARP inhibitors in combination therapy with camptothecins or temozolomide based on PARP trapping versus catalytic inhibition. J Pharmacol Exp Ther.

[30] O'sullivan CC, Moon DH, Kohn EC, Lee JM (2014). Beyond Breast and Ovarian Cancers: PARP Inhibitors for BRCA Mutation-Associated and BRCA-Like Solid Tumors. Front Oncol.

[31] Wick W, Weller M, van den Bent M, Sanson M, Weiler M, von Deimling A, Plass C, Hegi M, Platten M, Reifenberger G (2014). MGMT testing--the challenges for biomarker-based glioma treatment. Nat Rev Neurol.

[32] Zhang J, Stevens MF, Bradshaw TD (2012). Temozolomide: mechanisms of action, repair and resistance. Curr Mol Pharmacol.

[33] Bouwman P, Jonkers J (2014). Molecular pathways: how can BRCA-mutated tumors become resistant to PARP inhibitors?. Clin Cancer Res.

[34] Lord CJ, Ashworth A (2013). Mechanisms of resistance to therapies targeting BRCA-mutant cancers. Nat Med.

[35] Bunting SF, Callen E, Wong N, Chen HT, Polato F, Gunn A, Bothmer A, Feldhahn N, Fernandez-Capetillo O, Cao L, Xu X, Deng CX, Finkel T (2010). 53BP1 inhibits homologous recombination in Brca1-deficient cells by blocking resection of DNA breaks. Cell.

[36] Jaspers JE, Kersbergen A, Boon U, Sol W, van Deemter L, Zander SA, Drost R, Wientjens E, Ji J, Aly A, Doroshow JH, Cranston A, Martin NM (2013). Loss of 53BP1 causes PARP inhibitor resistance in Brca1-mutated mouse mammary tumors. Cancer Discov.

[37] Johnson N, Johnson SF, Yao W, Li YC, Choi YE, Bernhardy AJ, Wang Y, Capelletti M, Sarosiek KA, Moreau LA, Chowdhury D, Wickramanayake A, Harrell MI (2013). Stabilization of mutant BRCA1 protein confers PARP inhibitor and platinum resistance. Proc Natl Acad Sci U S A.

[38] Branzei D, Foiani M (2010). Maintaining genome stability at the replication fork. Nat Rev Mol Cell Biol.

[39] Wainberg ZA, Rafii S, Ramanathan RK, Mina LA, Byers LA, Chugh R, Goldman JW, Sachdev JC, Matei DE, Wheler JJ, Henshaw JW, Zhang C, Gallant G (14). Safety and antitumor activity of the PARP inhibitor BMN673 in a phase 1 trial recruiting metastatic small-cell lung cancer (SCLC) and germline BRCA-mutation carrier cancer patients. Journal of Clinical Oncology.

[40] Byers LA, Wang J, Nilsson MB, Fujimoto J, Saintigny P, Yordy J, Giri U, Peyton M, Fan YH, Diao L, Masrorpour F, Shen L, Liu W (2012). Proteomic profiling identifies dysregulated pathways in small cell lung cancer and novel therapeutic targets including PARP1. Cancer Discov.

[41] Smith MA, Reynolds CP, Kang MH, Kolb EA, Gorlick R, Carol H, Lock RB, Keir ST, Maris JM, Billups CA, Lyalin D, Kurmasheva RT, Houghton PJ (2015). Synergistic activity of PARP inhibition by talazoparib (BMN 673) with temozolomide in pediatric cancer models in the pediatric preclinical testing program. Clin Cancer Res.

[42] Erice O, Smith MP, White R, Goicoechea I, Barriuso J, Jones C, Margison GP, Acosta JC, Wellbrock C, Arozarena I (2015). MGMT Expression Predicts PARP-Mediated Resistance to Temozolomide. Mol Cancer Ther.

[43] Cardnell RJ, Feng Y, Diao L, Fan YH, Masrorpour F, Wang J, Shen Y, Mills GB, Minna JD, Heymach JV, Byers LA (2013). Proteomic markers of DNA repair and PI3K pathway activation predict response to the PARP inhibitor BMN 673 in small cell lung cancer. Clin Cancer Res.

[44] Rees MG, Seashore-Ludlow B, Cheah JH, Adams DJ, Price EV, Gill S, Javaid S, Coletti ME, Jones VL, Bodycombe NE, Soule CK, Alexander B, Li A (2016). Correlating chemical sensitivity and basal gene expression reveals mechanism of action. Nat Chem Biol.

[45] Hopkins TA, Shi Y, Rodriguez LE, Solomon LR, Donawho CK, DiGiammarino EL, Panchal SC, Wilsbacher JL, Gao W, Olson AM, Stolarik DF, Osterling DJ, Johnson EF (2015). Mechanistic Dissection of PARP1 Trapping and the Impact on In Vivo Tolerability and Efficacy of PARP Inhibitors. Mol Cancer Res.

[46] Lok BH, Gardner EE, Schneeberger VE, Ni A, Desmeules P, Rekhtman N, de Stanchina E, Teicher BA, Riaz N, Powell SN, Poirier JT, Rudin CM (2016). PARP Inhibitor Activity Correlates with SLFN11 Expression and Demonstrates Synergy with Temozolomide in Small Cell Lung Cancer. Clin Cancer Res.

[47] Tutt A, Robson M, Garber JE, Domchek SM, Audeh MW, Weitzel JN, Friedlander M, Arun B, Loman N, Schmutzler RK, Wardley A, Mitchell G, Earl H (2010). Oral poly(ADP-ribose) polymerase inhibitor olaparib in patients with BRCA1 or BRCA2 mutations and advanced breast cancer: a proof-of-concept trial. Lancet.

[48] Gelmon KA, Tischkowitz M, Mackay H, Swenerton K, Robidoux A, Tonkin K, Hirte H, Huntsman D, Clemons M, Gilks B, Yerushalmi R, Macpherson E, Carmichael J (2011). Olaparib in patients with recurrent high-grade serous or poorly differentiated ovarian carcinoma or triple-negative breast cancer: a phase 2, multicentre, open-label, non-randomised study. Lancet Oncol.

[49] Lord CJ, Tutt AN, Ashworth A (2015). Synthetic lethality and cancer therapy: lessons learned from the development of PARP inhibitors. Annu Rev Med.

[50] O'Connor MJ (2015). Targeting the DNA Damage Response in Cancer. Mol Cell.

[51] Garnett MJ, Edelman EJ, Heidorn SJ, Greenman CD, Dastur A, Lau KW, Greninger P, Thompson IR, Luo X, Soares J, Liu Q, Iorio F, Surdez D (2012). Systematic identification of genomic markers of drug sensitivity in cancer cells. Nature.

[52] Brenner JC, Feng FY, Han S, Patel S, Goyal SV, Bou-Maroun LM, Liu M, Lonigro R, Prensner JR, Tomlins SA, Chinnaiyan AM (2012). PARP-1 inhibition as a targeted strategy to treat Ewing's sarcoma. Cancer Res.

[53] Holbeck S, Chang J, Best AM, Bookout AL, Mangelsdorf DJ, Martinez ED (2010). Expression profiling of nuclear receptors in the NCI60 cancer cell panel reveals receptor-drug and receptor-gene interactions. Mol Endocrinol.

[54] Kummar S, Chen HX, Wright J, Holbeck S, Millin MD, Tomaszewski J, Zweibel J, Collins J, Doroshow JH (2010). Utilizing targeted cancer therapeutic agents in combination: novel approaches and urgent requirements. Nat Rev Drug Discov.

[55] Cong L, Ran FA, Cox D, Lin S, Barretto R, Habib N, Hsu PD, Wu X, Jiang W, Marraffini LA, Zhang F (2013). Multiplex genome engineering using CRISPR/Cas systems. Science.

[56] Chou TC (2010). Drug combination studies and their synergy quantification using the Chou-Talalay method. Cancer Res.

[57] Chou TC (2006). Theoretical basis, experimental design, and computerized simulation of synergism and antagonism in drug combination studies. Pharmacol Rev.

