# Supplementary material for: Resistance to PARP inhibitors by SLFN11 inactivation can be overcome by ATR inhibition
Source: Oncotarget. 2016 Sep 27;7(47):76534–50. doi: 10.18632/oncotarget.12266 (PMC5340226; doi:10.18632/oncotarget.12266)
Supplement: Supplementary file 1 [file oncotarget-07-76534-s001.pdf]

Resistance to PARP inhibitors by SLFN11 inactivation can be overcome by ATR inhibition

Supplementary Information

Junko Murai[1], Ying Feng[2], Sai-Wen Tang[1], Karen G. Yu[2], Kevin Ru[2], Yuqiao Shen[2], and Yves Pommier[1*]

Figure S1

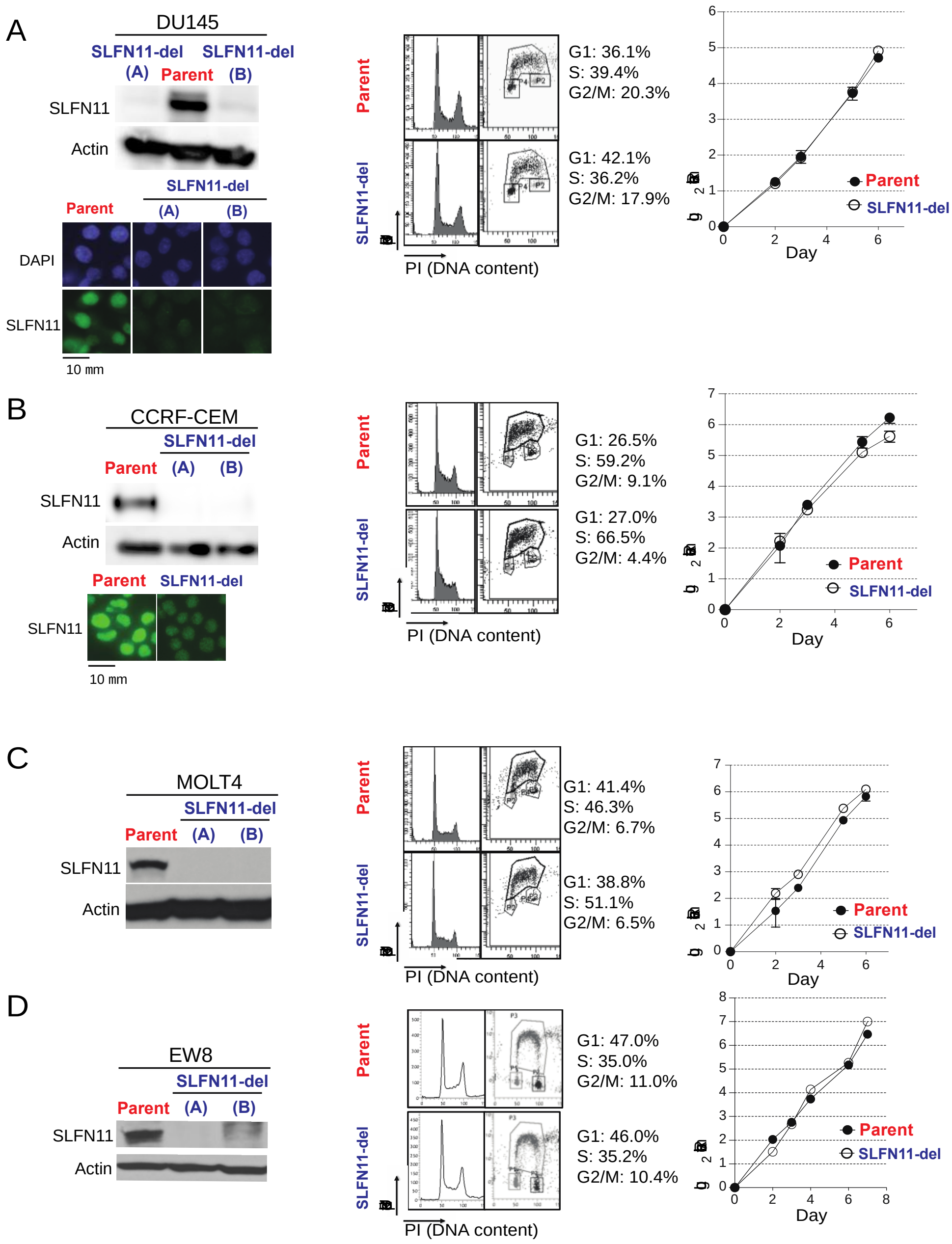

Supplementary Fig. S1. Generation and characterization of the *SLFN11*-deleted (*SLFN11*-del) cells in DU145, CCRF-CEM, MOLT4 and EW8 cell lines

A-D. Confirmation of *SLFN11* deletion in DU145 (A), CCRF-CEM (B), MOLT4 (C) and EW8 cells (D) by Western blotting using the indicated antibodies (A-D), and by immunofluorescence using anti-SLFN11 antibody (A and B). Two independent *SLFN11*-del clones targeted with different sequences by CRISPR/Cas9 system were generated for each cell line [*SLFN11*-del (A) and *SLFN11*-del (B)]. Cell cycle and cell growth analyses under normal condition are shown for each cell line (A-D). Percent population of individual cell phases (G1, S and G2) is annotated beside the cell cycle panels. The score is an average of three independent experiments. In the growth curve panels, the relative cell number of parental and *SLFN11*-del cells are plotted. Error bars represent SD (n ≥ 3).

Figure S2

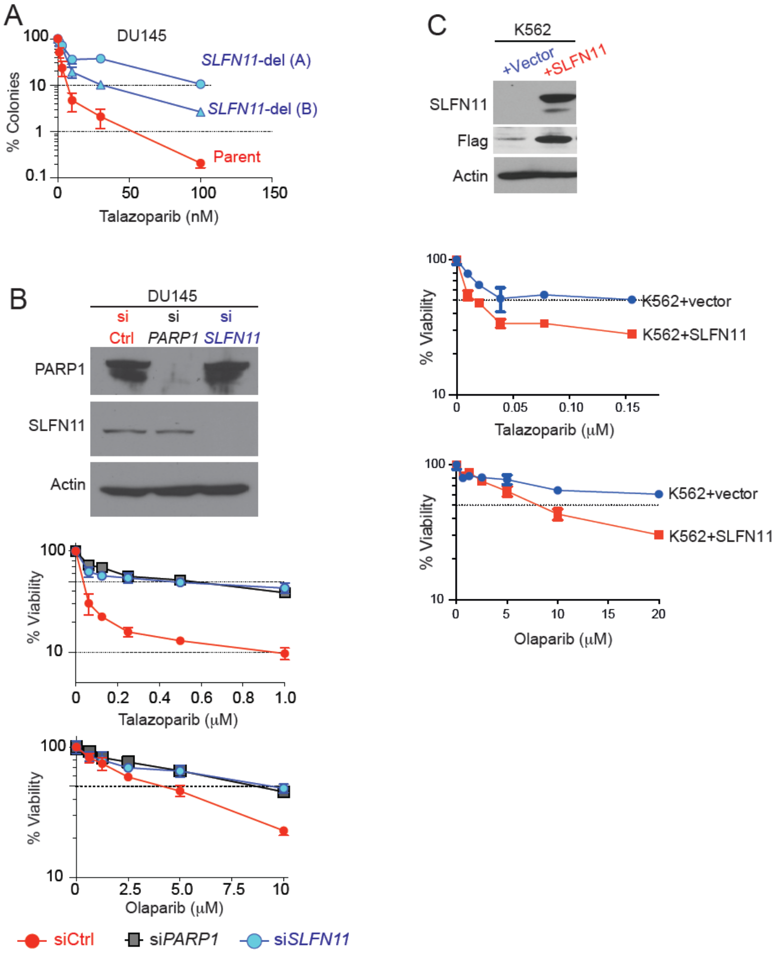

Supplementary Fig. S2. *SLFN11* inactivation confers resistance to talazoparib and olaparib

A. Colony formation assays with DU145 parental and *SLFN11*-del cells treated with the indicated concentrations of talazoparib. Colony number of untreated cells was set as 100%. Error bars represent standard deviation (SD, n = 3).

B. Control siRNA (siCtl), *SLFN11* siRNA (si*SLFN11*), and *PARP1* siRNA (si*PARP1*) were transfected into DU145 parental cells. Three days later, expression levels of each protein were analyzed by Western blotting with the indicated antibodies (upper). Two days after transfection, cells were treated with talazoparib or olaparib for an additional 72 hours. Viability was determined as Figure 1C. Error bars represent SD (n = 3).

C. Overexpression of SLFN11 in K562 cells was confirmed by Western blotting with the indicated antibodies (upper). K562 cells overexpressed Flag-tagged wild-type SLFN11 (+SLFN11) and vector only (+Vector) by lentiviral infection were treated with talazoparib (middle) or olaparib (lower) for 72 hours. Viability was determined as Figure 1C. Error bars represent SD (n=3)

Figure S3

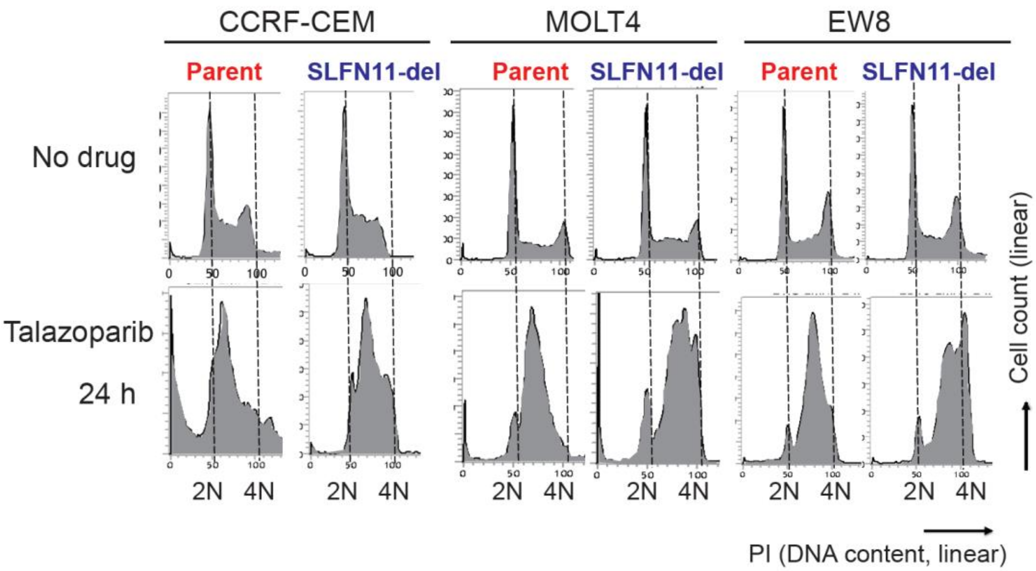

Supplementary Fig. S3. SLFN11-dependent S-phase arrest under talazoparib treatment

The indicated cells were continuously treated with 1 µM talazoparib for 24 hours, and then fixed and stained with propidium iodide (PI). Vertical dashed lines correspond to 2N and 4N DNA contents that are evaluated by PI staining.

Figure S4

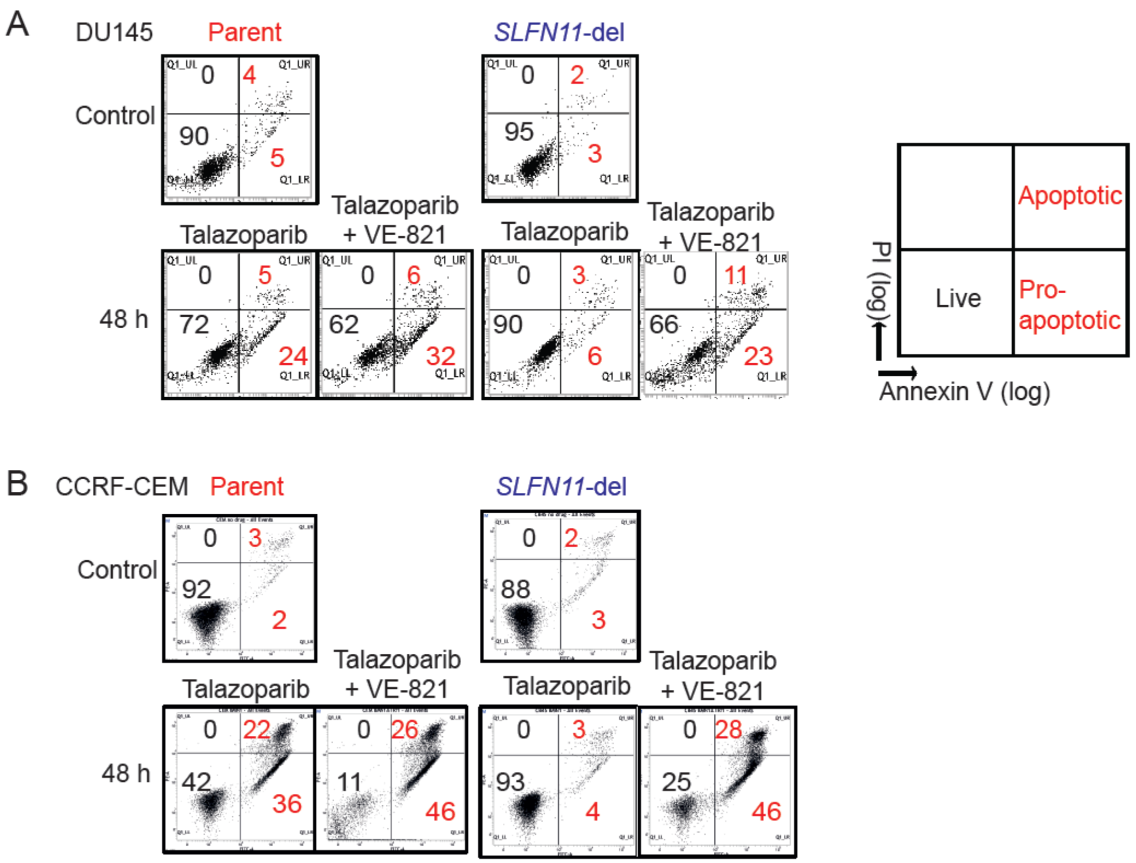

Supplementary Fig. S4. Addition of ATR inhibitor (VE-821) enhances apoptosis with talazoparib more in SLFN11-del cell than in the parental cells

A-B. Effect of talazoparib alone or talazoparib and ATR inhibitor (VE-821) combination on apoptotic cells 48 hours after continuous drug treatment. Annexin V/PI double staining was used to monitor apoptosis.

Figure S5

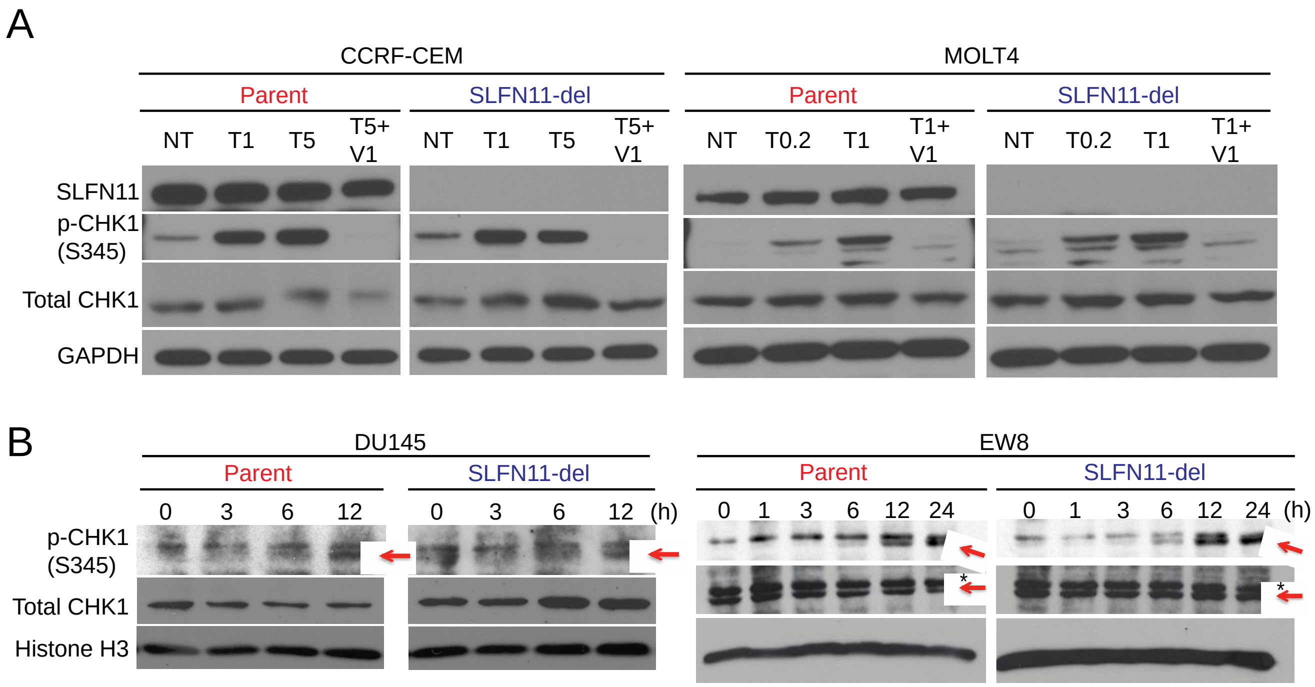

Supplementary Fig. S5. SLFN11 does not affect ATR activation

A-B. Western blot analyses of whole cell lysate prepared from the indicated cell lines. Blots were probed with the indicated antibodies. Anti-GAPDH (A) and anti-histone H3 (B) antibodies were used for loading control.

A. CCRF-CEM and MOLT4 cells were treated without drug (NT) or with talazoparib (T0.2: 0.2 μM, T1: 1 μM, T5: 5 μM)) or talazoparib plus VE-821 (ATR inhibitor) (V1: 1 μM) for 12 hours. The upregulation of phospho-CHK1 (Serine 345) signal was completely abolished by the addition of VE-821.

B. DU145 and EW8 cells were treated with 1 μM talazoparib for the indicated hours. Asterisks indicate non-specific bands, and red arrows indicate phospho-CHK1 (Serine 345).

Table S1: Combination Index (CI) in Figure 4B

	CI (combination index):		<0.1	very strong synergysm	
			0.1-0.3	strong synergysm	
			0.3-0.7	synergysm	
		**DU145 parent**		**DU145 *SLFN11*-del**	
Talazoparib (μM)	ATRi (μM)	Effect	CI	Effect	CI
0.625	1	0.947	0.10702	0.92	0.00606
1.25	1	0.962	0.11409	0.941	0.0039
2.5	1	0.962	0.22784	0.947	0.00335
5	1	0.965	0.39056	0.93	0.00499
Olaparib (μM)	ATRi (μM)	Effect	CI	Effect	CI
1.56	1	0.517	0.36524	0.4	0.47554
3.125	1	0.71	0.27165	0.62	0.16563
6.25	1	0.816	0.28464	0.747	0.09511
12.5	1	0.887	0.31562	0.846	0.05487
25	1	0.906	0.51104	0.857	0.08001
		**CCRF-CEM parent**		**CCRF-CEM *SLFN11*-del**	
Talazoparib (μM)	ATRi (μM)	Effect	CI	Effect	CI
0.3125	1	0.917	0.14175	0.846	0.05127
0.625	1	0.97	0.11209	0.943	0.02029
1.25	1	0.99	0.08524	0.961	0.01441
2.5	1	0.994	0.10926	0.967	0.01241
Olaparib (μM)	ATRi (μM)	Effect	CI	Effect	CI
1.56	1	0.56	0.42287	0.776	0.09073
3.125	1	0.861	0.30269	0.931	0.02518
6.25	1	0.98	0.15779	0.965	0.01344
12.5	1	0.992	0.17256	0.973	0.01083
25	1	0.995	0.25376	0.983	0.00723
		**MOLT4 parent**		**MOLT4 *SLFN11*-del**	
Talazoparib (μM)	ATRi (μM)	Effect	CI	Effect	CI
0.078	1	0.751	0.94225	0.73	0.50119
0.156	1	0.84	1.00987	0.838	0.42126
0.3125	1	0.935	0.76723	0.929	0.31465
0.625	1	0.984	0.41077	0.979	0.20641
1.25	1	0.9983	0.12643	0.9917	0.1548
Olaparib (μM)	ATRi (μM)	Effect	CI	Effect	CI
1.56	1	0.477	1.13129	0.555	0.92697
3.125	1	0.722	0.81124	0.802	0.66172
6.25	1	0.882	0.57674	0.91	0.53215
		**EW8 parent**		**EW8 *SLFN11*-del**	
Talazoparib (μM)	ATRi (μM)	Effect	CI	Effect	CI
0.25	1	0.946	0.53084	0.916	0.01099
0.5	1	0.965	0.38831	0.927	0.0088
1	1	0.982	0.17209	0.932	0.00787
Olaparib (μM)	ATRi (μM)	Effect	CI	Effect	CI
1.56	1	0.598	0.75657	0.61	0.42355

Table S2: Information of 36 small cell lung cancer cell lines for their sorce

Name	Vendor	Cat#	Name	Vendor	Cat#	Name	Vendor	Cat#
COR-L88	ECACC	92031917	NCI-H211	ATCC	CRL-5824	NCI-H1618	ATCC	CRL-5879
SBC-5	JCRB	JCRB0819	NCI-H2141	ATCC	CRL-5927	NCI-H1694	ATCC	CRL-5888
DMS 114	ATCC	CRL-2066	NCI-H2171	ATCC	CRL-5929	NCI-H1930	ATCC	CRL- 5906
DMS 79	ATCC	CRL-2049	NCI-H446	ATCC	HTB-171	NCI-H2081	ATCC	CRL-5920
NCI-H1836	ATCC	CRL-5898	NCI-H82	ATCC	HTB-175	SCLC-21H	CLS	300225
NCI-H1876	ATCC	CRL-5902	NCI-H889	ATCC	CRL-5817	NCI-H524	ATCC	CRL-5831
NCI-H1963	ATCC	CRL-5982	SHP-77	ATCC	CRL-2195	NCI-H526	ATCC	CRL-5811
NCI-H69	ATCC	HTB-119	NCI-H1105	ATCC	CRL-5856	NCI-H841	ATCC	CRL-5845
NCI-H1048	ATCC	CRL-5853	NCI-H2066	ATCC	CRL-5917	NCI-H2107	ATCC	CRL-5983
NCI-H1341	ATCC	CRL-5864	COR-L279	ECACC	96020724	NCI-H748	ATCC	CRL-5841
NCI-H146	ATCC	HTB-173	DMS-153	ATCC	CRL-2064			
NCI-H196	ATCC	CRL-5823	DMS-53	ATCC	CRL-2062			
NCI-H2029	ATCC	CRL-5913	NCI-H1092	ATCC	CRL-5855			
NCI-H209	ATCC	HTB-172	NCI-H1436	ATCC	CRL-5871			

ECACC: European Collection of Authenticated Cell Cultures
JCRB: Japanese Collection of Research Bioreso urces
ATCC: (company name)
CLS: CLS cell lines service

Table S3: Summary of IC_{50} for talazoparib single treatment, talazoparib + 10 μM temozolomide combination treatment, and SLFN11 and MGMT transcript in 36 small cell lung cancer cell lines. IC_{50}: inhibitory concentration 50%

Cell line	IC50 of talazoparib (nM)	IC50 of talazoparib (nM) in combination with 10 μM temozolomide	SLFN11 transcript (affymetrix value: log2)	MGMT transcript (affymetrix value: log2)
COR-L279	16.41	4.73	9.51	7.04
COR-L88	2000.00	2000.00	5.03	4.02
DMS-114	897.45	102.50	6.02	8.67
DMS153	5.31	3.24	9.03	8.31
DMS-53	46.51	0.02	6.36	4.79
DMS79	7.52	1.44	8.69	7.12
NCI-H1048	6.03	2.79	7.58	7.98
NCI-H1092	20.26	5.07	7.92	3.70
NCI-H1105	6.54	0.02	8.92	3.49
NCI-H1341	2000.00	2000.00	7.07	8.44
NCI-H1436	2000.00	2000.00	3.71	9.38
NCI-H146	12.07	0.55	4.43	6.45
NCI-H1618	29.79	0.02	8.77	4.05
NCI-H1694	34.85	8.19	7.89	7.44
NCI-H1836	2000.00	2000.00	3.78	3.58
NCI-H1876	23.55	4.09	8.98	7.67
NCI-H1930	43.51	8.55	6.51	8.34
NCI-H1963	345.75	20.04	3.66	6.96
NCI-H196	2000.00	2000.00	3.79	8.14
NCI-H2029	2000.00	773.71	4.37	7.02
NCI-H2066	2000.00	2000.00	3.64	8.28
NCI-H2081	18.35	0.40	7.48	6.79
NCI-H209	10.41	1.06	8.79	6.44
NCI-H211	7.67	2.12	4.12	6.43
NCI-H2141	41.66	8.64	6.86	5.20
NCI-H2171	18.08	4.47	4.14	7.52
NCI-H446	13.71	0.02	3.85	4.21
NCI-H524	8.93	1.74	3.70	7.07
NCI-H526	39.81	3.91	7.84	7.76
NCI-H69	22.63	0.12	3.98	4.11
NCI-H82	97.44	9.20	3.62	8.06
NCI-H841	2000.00	681.23	3.87	8.59
NCI-H889	35.49	4.54	3.81	7.42
SBC-5	178.19	13.77	3.93	9.90
SCLC-21H	53.70	6.37	3.73	7.22
SHP-77	2000.00	2000.00	3.67	3.81
